# Organoids from human tooth showing epithelial stemness phenotype and differentiation potential

**DOI:** 10.1007/s00018-022-04183-8

**Published:** 2022-02-26

**Authors:** Lara Hemeryck, Florian Hermans, Joel Chappell, Hiroto Kobayashi, Diether Lambrechts, Ivo Lambrichts, Annelies Bronckaers, Hugo Vankelecom

**Affiliations:** 1 Laboratory of Tissue Plasticity in Health and Disease, Cluster of Stem Cell and Developmental Biology, Department of Development and Regeneration, Leuven Stem Cell Institute, KU Leuven (University of Leuven), Leuven, Belgium; 2 Faculty of Medicine and Life Sciences, Biomedical Research Institute (BIOMED), UHasselt (Hasselt University), Diepenbeek, Belgium; 3 Department of Bioinformatics, Bit.bio, Babraham Research Campus, Cambridge, UK; 4 Department of Anatomy and Structural Science, Faculty of Medicine, Yamagata University, Yamagata, Japan; 5 VIB – Center for Cancer Biology, Leuven, Belgium; 6 Laboratory for Translational Genetics, Department of Human Genetics, KU Leuven, Leuven, Belgium

**Keywords:** Tooth, Organoids, Stem cells, Ameloblasts, Assembloids, TGFβ

## Abstract

Insight into human tooth epithelial stem cells and their biology is sparse. Tissue-derived organoid models typically replicate the tissue’s epithelial stem cell compartment. Here, we developed a first-in-time epithelial organoid model starting from human tooth. Dental follicle (DF) tissue, isolated from unerupted wisdom teeth, efficiently generated epithelial organoids that were long-term expandable. The organoids displayed a tooth epithelial stemness phenotype similar to the DF’s epithelial cell rests of Malassez (ERM), a compartment containing dental epithelial stem cells. Single-cell transcriptomics reinforced this organoid-ERM congruence, and uncovered novel, mouse-mirroring stem cell features. Exposure of the organoids to epidermal growth factor induced transient proliferation and eventual epithelial-mesenchymal transition, highly mimicking events taking place in the ERM in vivo. Moreover, the ERM stemness organoids were able to unfold an ameloblast differentiation process, further enhanced by transforming growth factor-β (TGFβ) and abrogated by TGFβ receptor inhibition, thereby reproducing TGFβ's known key position in amelogenesis. Interestingly, by creating a mesenchymal-epithelial composite organoid (assembloid) model, we demonstrated that the presence of dental mesenchymal cells (i.e. pulp stem cells) triggered ameloblast differentiation in the epithelial stem cells, thus replicating the known importance of mesenchyme-epithelium interaction in tooth development and amelogenesis. Also here, differentiation was abrogated by TGFβ receptor inhibition. Together, we developed novel organoid models empowering the exploration of human tooth epithelial stem cell biology and function as well as their interplay with dental mesenchyme, all at present only poorly defined in humans. Moreover, the new models may pave the way to future tooth-regenerative perspectives.

**Supplementary Information:**

The online version contains supplementary material available at 10.1007/s00018-022-04183-8.

## Introduction

Teeth play essential roles in food mastication and speech. Moreover, tooth physiology is more and more highlighted to impact body health and disease [1–3]. In contrast to the wealth of knowledge on tooth development, homeostatic maintenance and repair in rodents, tooth biology remains far from understood in humans [4]. Although stem cells of the mesenchymal compartments such as dental pulp and periodontal ligament (PDL) have substantially been characterized, knowledge on human tooth epithelial stem cells regarding presence, phenotype and biological function is scarce [4]. Some indications for their existence have been found in the epithelial cell rests of Malassez (ERM), a network of epithelial cells that is present in the dental follicle (DF) which encloses unerupted teeth and upon tooth eruption remains present in the PDL around the root [5]. These nests of epithelial cells express some stem cell-associated markers, and may play a role in regeneration of enamel and PDL following injury and inflammation, although repair capacity appears limited in postnatal life [5, 6]. During tooth development, enamel is formed by epithelial cells called ameloblasts [4]. It has been reported that ERM-derived cells, when co-cultured with dental pulp stem cells (DPSCs), can differentiate into ameloblast-like cells [7]. However, 2D-cultured ERM cells show highly limited growth capacity and rapid loss of phenotype [8–12].

A powerful method to in vitro grow and expand tissue epithelial stem cells is provided by the organoid technology [13–17]. Organoids are 3D cell constructs that self-develop by proliferative expansion from tissue’s epithelial stem cells when the dissociated primary tissue sample (containing the stem cells as single cells or contained within cell clusters) is seeded into an extracellular matrix (ECM)-mimicking scaffold (typically, Matrigel) and cultured in a defined cocktail of growth factors replicating stem cell niche signaling (if known) and/or tissue embryogenesis. Among others, activation of wingless-type MMTV integration site (WNT) and epidermal growth factor (EGF) signaling are typically needed [14–16]. Resultant organoids duplicate the epithelial stem cell compartment of the tissue of origin in molecular phenotype and functional characteristics, and can generate differentiated tissue cell types under specified culture conditions [13–15]. As an important asset, organoid cultures can be serially expanded (passaged) without loss of characteristics, thereby providing a robust and faithful source of the primary tissue’s epithelial stem cells and overcoming their generally limited availability and culture-ability. Typically, epithelial organoid models are established without the need for prior isolation of the epithelial (stem) cells from the dissociated whole-tissue sample since the accompanying mesenchymal cells do not thrive in the specific culture conditions used and are swiftly lost at culture and passaging [13, 14, 16, 17]. Although meanwhile derived from numerous organs, epithelial organoids have not been established yet from human tooth [18, 19]. A previous study reported that ERM cells, seeded in Matrigel, grew as ‘organoids’ [8]. However, these structures were not deeply characterized and did not adhere to the current hallmarks of tissue-derived organoids [13–17] such as (clonal) derivation and expansion from epithelial tissue stem cells under WNT-promoting conditions, and robust and long-term expandability. Other studies described the construction of bioengineered 3D dental structures, however, only from animal origin (mouse, rat, dog, pig) at embryonic or neonatal age and non-expandable [18, 20–22].

Here, we report the successful establishment of long-term expandable organoid cultures starting from human tooth (i.e. from the DF of third molars). The organoids show epithelial stemness characteristics mirroring ERM stem cells, and display ameloblast differentiation property reinforced by the presence of TGFβ or dental mesenchymal cells, thereby recapitulating ERM/dental epithelial stem cell (DESC) features and known in vivo processes. The new organoid models provide a valuable research tool to explore human tooth epithelial stem cell biology and epithelium–mesenchyme interplay, at present only poorly understood, thereby paving the way to unraveling their roles in tooth homeostasis and potential repair. Moreover, the tractable biological tooth stem cell structures represent an appealing step toward dental regenerative replacement prospects.

## Results

### Organoids can be established from human dental follicle

**Fig. 1:**
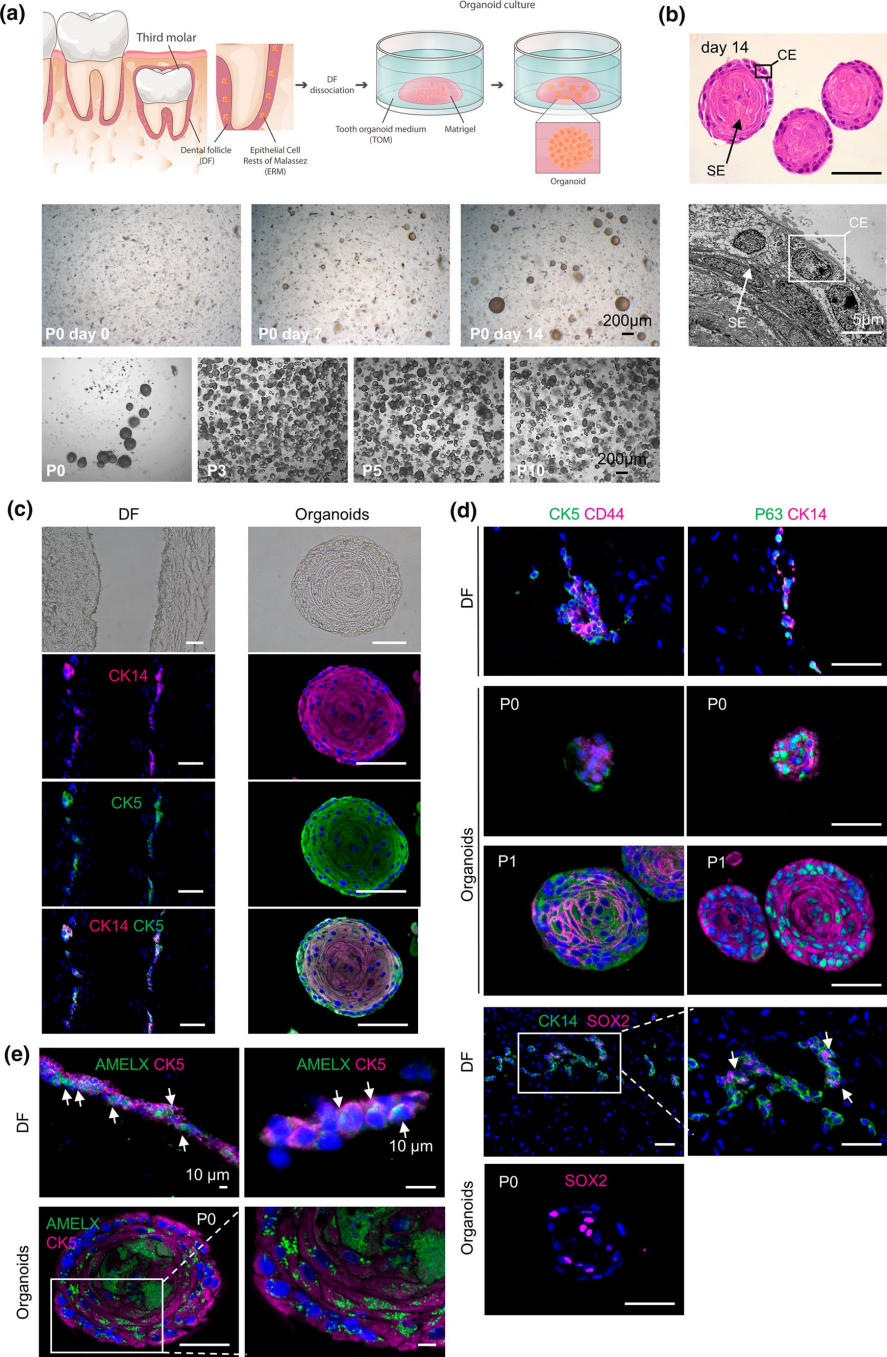
Establishment of organoids from human dental follicle. **a** Schematic of organoid culture set-up. Progressing development of organoid structures after seeding dissociated dental follicle (DF) in tooth organoid medium (TOM) (passage 0, P0), and robust passageability (brightfield pictures of indicated P). **b** Histological (H&E) and ultrastructural (TEM) analyses of tooth organoids grown in TOM for 14 days. Box and arrow indicate cuboidal epithelium (CE) and squamous epithelium (SE), respectively. **c-e** Brightfield phase-contrast images and immunofluorescence staining pictures for markers as indicated, of primary DF tissue and full-grown (day-14) organoids. Arrows indicate double-positive cells of indicated markers. Boxed areas are enlarged. DAPI (blue) was used to label nuclei. Scale bars: 50 µm, unless indicated otherwise

To develop epithelial organoids starting from human tooth, the DF, known not only to encompass a large mesenchymal component but also the small epithelial ERM compartment [5], was isolated from unerupted third molars (wisdom teeth) extracted from adolescent patients (Fig. 1a). After tissue dissociation, the epithelial–mesenchymal cell mixture, comprising single cells and cell clusters, was embedded in Matrigel and cultured in a precisely defined medium. Organoids are typically established using a cocktail of growth and regulatory factors active in the tissue’s epithelial stem cell niche. In case niche signals are unresolved, factors with a key role in the tissue’s embryonic development are applied [13–17]. Hence, since the human tooth epithelial stem cell niche is undefined, we tested growth and signaling factors shown to play a role in tooth development, including sonic hedgehog (SHH), fibroblast growth factors (FGFs) and insulin-like growth factor-1 (IGF1) [23, 24]. We started with a medium containing these factors as well as generic organoid growth factors (such as WNT activators, nicotinamide, BMP inhibitor and p38 MAPK inhibitor) [13–16, 25] and assessed the essentiality of individual factors by omitting. The BMP inhibitor (Noggin), p38 MAPK inhibitor (SB202190), WNT activator (R-spondin 1 (RSPO1)), IGF1, SHH, nicotinamide, and FGFs (FGF2, FGF8, FGF10) were all evidently needed for efficient organoid formation (Supplementary Fig. 1a). Eventually, this systematic evaluation led to defining an optimized medium (further referred to as tooth organoid medium or TOM; Supplementary Table 1), allowing to develop organoid lines from DF samples at 100% efficiency (Supplementary Table 2).

Organoid structures gradually developed in 2 weeks’ time (Fig. 1a; passage 0 (P0)), growing out of cell clusters (typically 4–8 cells wide by 20 cells long, similar to the ERM cell groups present in the primary DF tissue; Supplementary Fig. 1b,c) [5, 26], or of single cells, indicating the capability of clonal development (Supplementary Fig. 1b). Importantly, the organoids were amenable to long-term expansion, at present for more than 10 consecutive passages (i.e. 5 months) (Fig. 1a). At passaging, the organoids were dissociated into single cells and organoid structures efficiently re-grew. When starting from a mixture of cells dissociated from either eGFP^+^ or eGFP^−^ organoid cultures, the organoids that reformed were homogeneously fluorescent or non-fluorescent, suggesting clonal regrowth at passaging (Supplementary Fig. 1d). Mesenchymal cells, also present in the dissociated DF cell mixture, adhered to the bottom of the culture plate following sample seeding (P0; Supplementary Fig. 1e), and were swiftly lost at passaging in the standard, epithelial-favoring organoid culture conditions used (P1; Supplementary Fig. 1e). During a single-passage 14-day culture period, organoids progressively increased in size while the proportion of proliferating (KI67^+^) cells gradually decreased and the fraction of apoptotic (cleaved-caspase 3, CC3^+^) cells slightly enhanced, although to only low levels which remained invariable over different passages (as determined in full-grown day-14 organoids) (Supplementary Fig. 1f,g). Full-grown organoid size also remained constant over passaging, after a first significant increase from P0 to P1 (Supplementary Fig. 1g). Within individual passages, the organoids displayed considerable size homogeneity (Supplementary Fig. 1g). Finally, organoid cultures could be reconstituted after cryopreservation, and were also establishable from the DF of already erupted wisdom teeth (Supplementary Fig. 1h).

The developed organoid structures displayed a dense morphology (Fig. 1a,b), showing an outer border of stratified cuboidal epithelium (CE) with cells displaying a high nucleo-cytoplasmic ratio, and an adjoining stratified squamous epithelium (SE; Fig. 1b). In DF tissue, epithelial cells with high nucleo-cytoplasmic ratio are present in the ERM (Supplementary Fig. 1c) [5]. In further analogy, the ERM markers cytokeratin (CK) 14 and CK5 [27] were detected in the organoids, as they are observed in compartments of the original DF tissue (Fig. 1c). Importantly, the mesenchymal (fibroblast) marker CD90 (Thy-1 cell surface antigen, THY1), which is observed in compartments of the original DF tissue was not detected in the organoids (Supplementary Fig. 1i), indicating the absence of pure mesenchymal cells in the (epithelial) organoids. Previous studies proposed that the ERM contains DESCs, among others marked by CD44 and P63 [12, 27, 28]. Interestingly, these markers, indeed observed in the primary DF tissue (Fig. 1d), were also detected in the derived organoids, both at initial formation (P0) and after passaging (P1; Fig. 1d). Moreover, the organoids and the native DF tissue expressed SOX2 (Fig. 1d), a well-known marker of DESCs in mouse [29–32] and detected in epithelium of developing teeth in humans [4]. Finally, the organoids showed prominent gene expression of the proposed ERM stem cell marker integrin-α6 (*ITGα6*) [8, 28], as well as of factors playing an important role in embryonic development of the dental epithelium (i.e. paired like homeodomain 2 (*PITX2*) [33] and bone-morphogenetic protein 4 (*BMP4*)) [34], all markers also detected in the original DF tissue (Supplementary Fig. 1j). Finally, we also observed expression of amelogenin (AMELX) in both primary tissue (more in particular in the CK5^+^ ERM region of the DF) and derived organoids (Fig. 1e). AMELX is not only a main constituent of enamel matrix but also a suggested marker of ERM cells (described in rat) [6, 35–37]. Proposed functions of this ERM-produced AMELX include maintenance of PDL space [38] and stimulation of cementoblast differentiation [39, 40]. Of note, AMELX localization can show a punctuated pattern, being present in vesicles and/or secreted in the extracellular space [41], which is also observed here (Fig. 1e). 

Taken together, epithelial organoids can be established from human tooth-derived DF, displaying an ERM-mirroring, stemness expression phenotype and possessing robust long-term expandability.

### Single-cell transcriptomics reinforces the organoid-ERM congruence

**Fig. 2:**
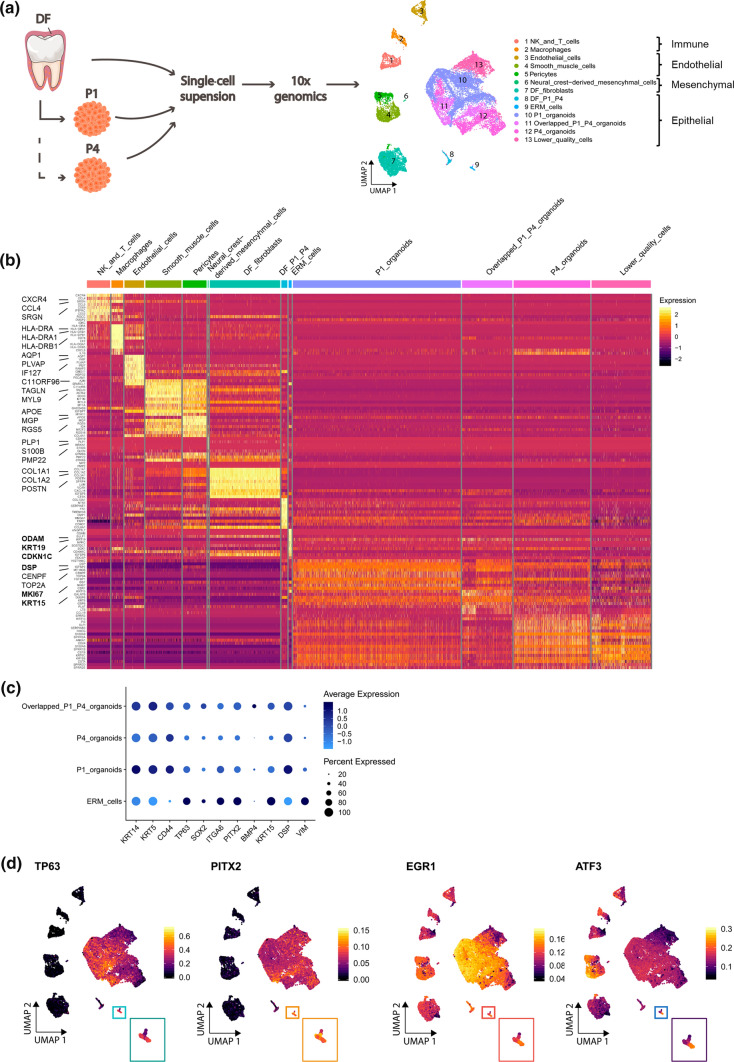

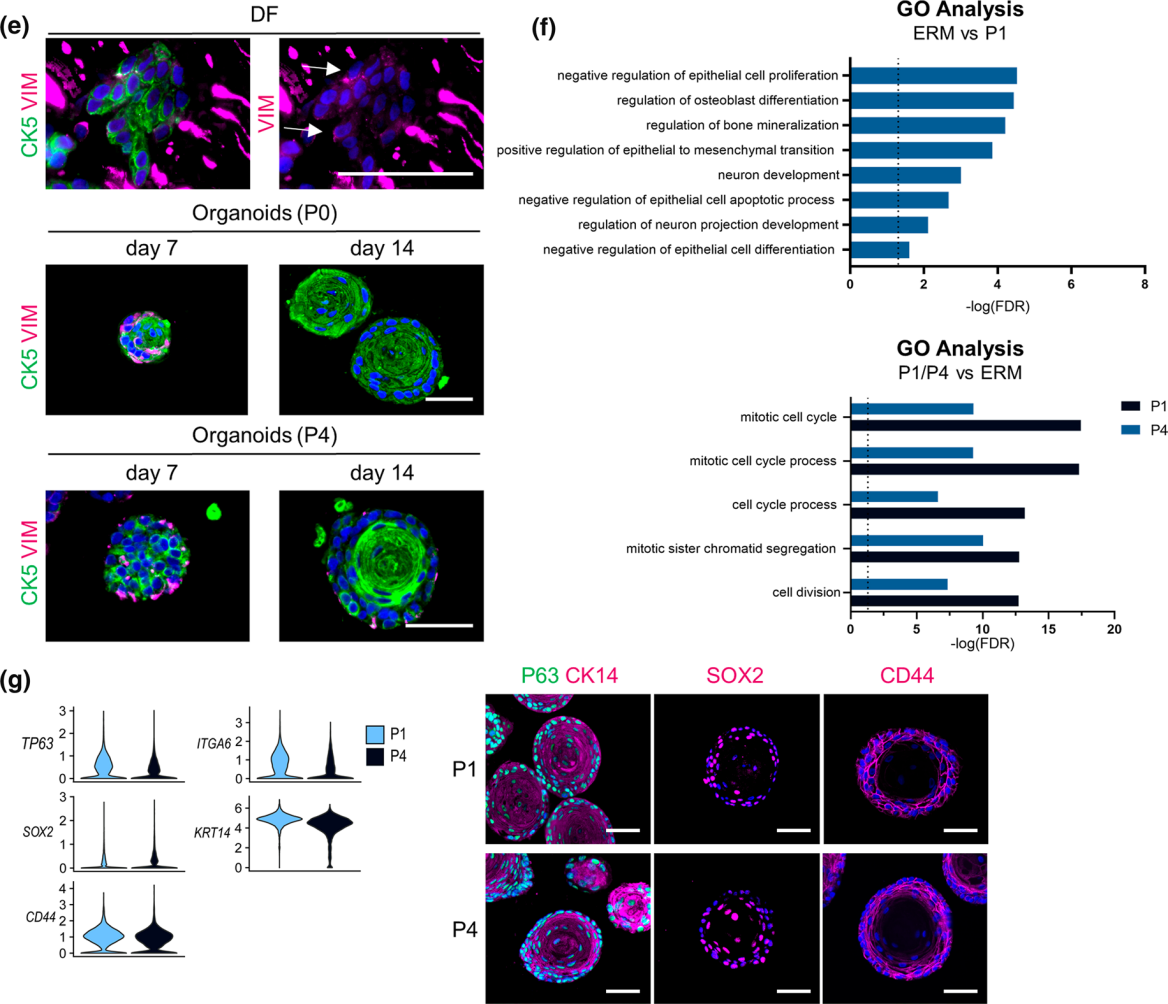
Single-cell transcriptomic profiling of primary DF and corresponding organoids. **a** Experimental overview of the scRNA-seq analysis. UMAP plot of the annotated cell clusters in the integrated DF-organoid dataset. DF, dental follicle; ERM, Epithelial Cell Rests of Malassez; NK, natural killer cells. **b** Heatmap displaying the scaled expression of the top 10 differentially expressed genes (DEGs) per cluster. Genes specifically described in the text are highlighted in bold. **c** Dot plot displaying the percentage of cells (dot size) expressing indicated marker genes with average expression levels (color intensity) (see scales) in the ERM and organoid clusters. **d** Indicated regulons projected on UMAP plot. The ERM cluster is magnified at the bottom. **e** Immunofluorescence staining for markers as indicated in primary DF tissue and organoids at specified time points. DAPI (blue) was used to label nuclei. **f** Significant (FDR ≤ 0.05) DEG-based GO terms enriched in ERM *versus* P1 organoids (top) or in P1 and P4 organoids together *versus* ERM (bottom). **g** Violin plots showing gene expression level of indicated stemness markers in P1 and P4 organoids. Immunofluorescence staining of P1 and P4 organoids for the indicated markers. DAPI (blue) was used to label nuclei. Scale bars: 50 µm

To decode the organoids in more granular detail, we applied single-cell RNA-sequencing (scRNA-seq) analysis on DF-derived organoids (at P1 and P4) together with their primary tissue (Fig. 2a; Supplementary Table 2). Unsupervised clustering of the aggregate data and visualization using uniform manifold approximation and projection (UMAP) [42] exposed main DF cell-type clusters annotated using reported markers [43] comprising immune, endothelial, mesenchymal and epithelial compartments, and grouped organoid clusters with noticeable overlap of the P1 and P4 cultures (Fig. 2a; Supplementary Fig. 2a; Supplementary Dataset 1). Of note, a cluster of lower quality cells (i.e. with low gene counts) was also discerned (Fig. 2a), not filtered out using the applied quality thresholds (see Methods; Supplementary Fig. 2b). This cluster likely comprises dying cells from the organoids’ core, as supported by gene ontology (GO) analysis [44] revealing an enriched ‘cell death’ biological term (Supplementary Fig. 2c), which conforms to ultrastructural features of apoptotic nuclei and absence of cell organelles in the organoids’ center (Supplementary Fig. 2c).

Compiling the clusters’ top 10 differentially expressed genes (DEGs; Supplementary Dataset 1) in a heatmap exposed specific expression patterns of the different clusters (Fig. 2b). Several of the reported stem cell and ERM markers (see above and [12, 27, 28, 33–35]) were found expressed in the organoids (Fig. 2c; Supplementary Fig. 2a,d), thus corroborating our observations above on organoid-ERM correspondence. Of note, mesenchymal markers such as fibroblast activation protein alpha (*FAP*) and collagen type I alpha 1 chain (*COL1A1*), being present in the DF mesenchymal (fibroblast) cluster, were not detected in the organoid clusters, thereby again demonstrating the absence of pure mesenchymal cells in the (epithelial) organoids (Supplementary Fig. 2d). In further analogy with the transcriptomic organoid-ERM congruence, gene-regulatory network (regulon) analysis using pySCENIC [45] (i.e. defining core transcription factors with their positively regulated target genes in single cells) showed high TP63 and PITX2 regulon activity in both organoids and ERM (Fig. 2d). Predicted target genes of the PITX2 regulon include *SOX2*, *TP63*, *PITX2*, *KRT5*, *KRT14*, and *BMP4* (Fig. 2c). Interestingly, the newly proposed mouse incisor epithelial (stem) cell markers KRT15 and dentin sialoprotein (DSP) [46] were among the top 10 DEGs in the organoid as well as ERM clusters (Fig. 2b,c; Supplementary Fig. 2e). Moreover, high regulon activity of early growth response 1 (EGR1) (marking a transient progenitor population in mouse incisor [43]) was observed in organoid clusters and ERM, while substantial regulon activity of activating transcription factor 3 (ATF3) (recently reported as new mouse incisor epithelial cell marker [46]) also showed up in the organoid and ERM clusters (Fig. 2d). Together, our single-cell transcriptomic analysis uncovered new, mouse-mirroring molecular features for human ERM, at present ill-defined. Intriguingly, we also detected gene expression of the mesenchymal marker vimentin (*VIM*) in ERM cells (Fig. 2c; Supplementary Fig. 2a,e). In agreement, and in analogy with a previous report [47], we observed co-expression of VIM in ERM (CK5^+^) cells of the primary DF tissue (Fig. 2e), which was recapitulated in the initiating organoids (Fig. 2e; P0, day 7). These findings may point to a hybrid epithelial/mesenchymal (E/M) nature which has recently been correlated with stemness and active stem cells in several other (developing) tissues (such as human fetal pituitary, intestine, liver, lung) [48, 49]. VIM expression faded during further organoid culturing (Fig. 2e; day 14). However, VIM expression transiently reiterated at each passaging (as shown for P4; Fig. 2e), suggesting that the hybrid E/M (active stem cell) phenotype is re-activated at re-seeding. Taken together, our profound single-cell transcriptomic analysis further reinforces the congruence between the DF-derived organoids and the ERM (stem) cells residing in this tooth tissue. To finally corroborate this relationship, we isolated ERM stem cells from DF tissue by FACS based on ITGα6 expression ([8, 28] and Supplementary Fig. 2f), and seeded the cells in organoid-developing conditions. The ITGα6^+^ ERM cells formed organoids whereas ITGα6^−^ cells did not (Supplementary Fig. 2f). Interestingly, the latter culture only showed mesenchymal cells adhering to the bottom of the culture plate which were not observed in the ITGα6^+^ cell culture (Supplementary Fig. 2f), further supporting that the DF’s mesenchymal cells do not contribute to, or make part of, organoid growth.

We further compared the organoids with the ERM by applying GO analysis. Negative regulation of ‘epithelial cell proliferation’, of ‘epithelial cell apoptotic process’ and of ‘epithelial cell differentiation’ are significantly enriched in the ERM versus the organoids (P1) (Fig. 2f; Supplementary Dataset 2, 3, 4a,b) which is in line with the quiescence stemness character of ERM under homeostatic conditions as reported before [5, 7, 12]. For instance, the cell cycle inhibitor *CDKN1C* is among the top DEGs in the ERM cluster (Fig. 2b). On the other hand, GO terms associated with cell cycle division are enriched in the organoids when compared to ERM (Fig. 2f; Supplementary Dataset 2, 3, 4c,d). In analogy, gene expression of the proliferation marker *MKI67* is prominent in the organoid clusters and absent in the ERM cluster, further corroborated by immunostaining (Fig. 2b; Supplementary Fig. 2g). Also, other proliferation markers such as topoisomerase II alpha (*TOP2A*) and centromere protein F (*CENPF*) were found almost exclusively expressed in the organoid clusters (Supplementary Fig. 2g). Interestingly, *CENPF* was recently discovered in the dental epithelium of the continuously growing mouse incisor [46]. Together, these data provide supportive evidence that the ERM stem cells, being quiescent in vivo, are proliferatively (re-)activated in organoid culture.

In further GO analysis, we found that the biological terms ‘regulation of osteoblast differentiation’, ‘regulation of bone mineralization’ and ‘regulation of neuron projection development/neuron development’ are enriched in the ERM (Fig. 2f; Supplementary Dataset 2, 3, 4a,b), all representing previously proposed biological functions of this specific DF cell compartment [5, 26, 50]. Finally, the higher mesenchymal character of the ERM as compared to full-grown organoids (Fig. 2c,e) is reflected in the enriched ‘positive regulation of epithelial-mesenchymal transition’ (EMT) term (Fig. 2f), in agreement with higher regulon activity of the EMT-driving transcription factors TWIST1 and ZEB1 in the ERM (Supplementary Fig. 2h; Supplementary Dataset 2, 3, 4a,b).

In general, gene expression signatures of P1 and P4 organoids display substantial similarity (Fig. 2b; Supplementary Dataset 5). In particular, expression of stemness markers remains comparable after the additional passaging (Fig. 2g), indicating that the tooth organoids retain their stemness phenotype during expansive culture.

Taken together, our detailed scRNA-seq interrogation demonstrates and reinforces the organoid-ERM stemness relationship and uncovered new molecular fingerprints of human ERM, at present only poorly defined.

### EGF induces a proliferative and EMT phenotype in tooth organoids, reminiscent of in vivo events in the ERM

To establish organoids from primary tissues, supplementation of EGF is generally found quintessential [13–16]. Hence, it is remarkable that DF-derived organoids develop and expand in the absence of exogeneous EGF (TOM; Supplementary Table 1). scRNA-seq mining exposed that the EGF receptor (EGFR) ligands amphiregulin (*AREG*) and heparin-binding EGF (*HB-EGF*), together with *EGFR*, are highly expressed in the organoids (Supplementary Fig. 3a) which may substitute for EGF. In line, blocking the EGFR with cetuximab (added at passaging) compromised organoid growth, resulting in smaller organoids (Supplementary Fig. 3b), thereby revealing the need for endogenous EGFR signaling.

**Fig. 3:**
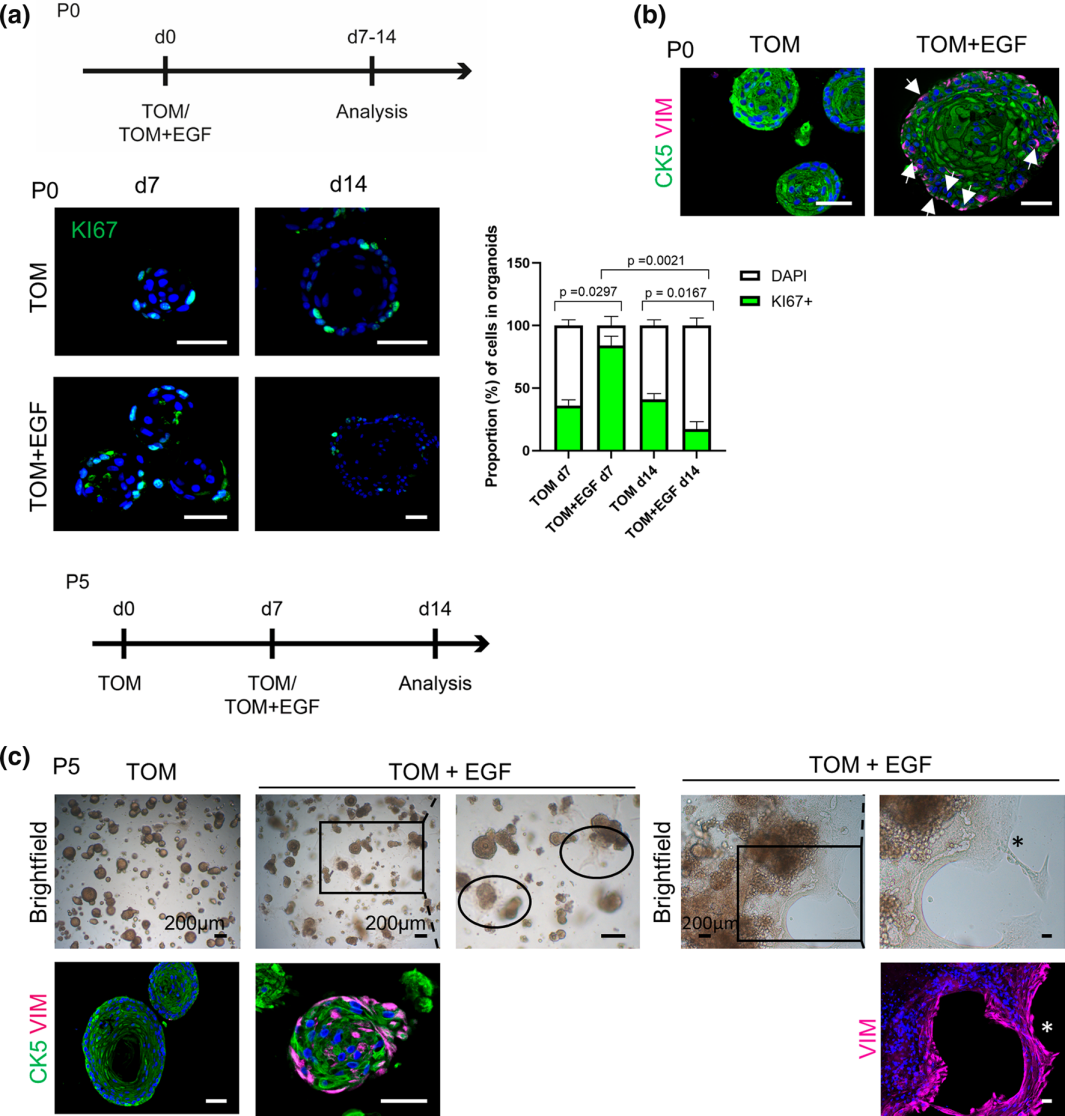
Effect of EGF on tooth organoid culture. **a** Timeline of experimental set-up (d, day). Immunofluorescence analysis and quantification of KI67^+^ cells (mean ± SEM; *n* = 3 biological replicates) in organoids cultured as indicated. DAPI (blue) was used to label nuclei. **b** Immunofluorescence staining for the indicated markers of full-grown organoids (day 14; P0) cultured in medium as denoted. Arrows indicate double VIM^+^CK5^+^ cells. DAPI (blue) was used to label nuclei. **c** Timeline of experimental set-up. Left part: brightfield pictures of organoid cultures (day 14) as indicated. Boxed area is enlarged. Encircled areas show cell growth at the bottom of the culture plate. Immunofluorescence staining of full-grown organoids (day 14; P5) cultured as indicated for the indicated markers. Right part: brightfield pictures and immunofluorescence (VIM) staining of cells grown at the bottom of the plate (day 14; P5). Boxed area is enlarged. DAPI (blue) was used to label nuclei. Asterisk mark for orientation. Scale bars: 50 µm, unless indicated otherwise

In vivo, it is known that elevated EGF levels in the ERM compartment (as, for instance, occurring upon tooth movement, infection or trauma) activates ERM cell proliferation [5, 26, 51]. Supplementation of EGF (50 ng/ml) to initiating organoid cultures (medium referred to as TOM + EGF) resulted in an increased number of proliferating (KI67^+^) cells in the organoids (P0; day 7; Fig. 3a). The increase was swiftly followed by a decline in proliferation (day 14; Fig. 3a), coinciding with the induction of an EMT process, as supported by the emergence of VIM expression in the organoids’ border, found colocalized with epithelial CK5 or P63 in several cells (Fig. 3b; Supplementary Fig. 3c). Moreover, exposing the organoids to EGF after preceding growth and expansion in TOM led to a same process with appearance of VIM^+^ cells (Fig. 3c), as well as migration of cells out of the organoid structures to grow at the bottom of the culture plate, displaying mesenchymal (spindle-like) morphology and VIM expression (Fig. 3c). This motile behavior further underscores the occurrence of EMT, also supported by the upregulated expression of specific mesenchymal/EMT-linked factors (Supplementary Fig. 3d). It has been proposed that EMT induction in the ERM (as, for instance, occurring upon damaging tooth impact) enables the cells to migrate and eventually contribute to regeneration of neighboring tissues [5, 26, 47].

Taken together, adding EGF to the organoids recapitulates functional in vivo behavior of the ERM, thus advancing our new tooth organoid model as an interesting tool to study ERM phenotype and conduct, to date not well understood.

### The tooth organoids are amenable to an ameloblast differentiation process

During tooth development, DESCs give rise to ameloblasts which produce enamel matrix proteins (EMPs) for amelogenesis [4, 52, 53]. It has also been shown that ERM can differentiate into ameloblast(-like) cells [6, 7] and produce EMPs [35, 54]. Ameloblast differentiation encompasses a secretory stage with production of the EMPs AMELX and ameloblastin (AMBN), and a maturation stage during which amelotin (AMTN) and odontogenic-ameloblast associated protein (ODAM) are produced [4, 35, 52, 53]. The EMPs are proteolytically cleaved by matrix metalloproteinase 20 (MMP20) and kallikrein (KLK4), typically expressed during the secretory and maturation phase, respectively. Here, we examined whether the DF-derived organoids, possessing an epithelial ERM-stemness phenotype, can be driven into differentiation toward ameloblasts.

**Fig. 4:**
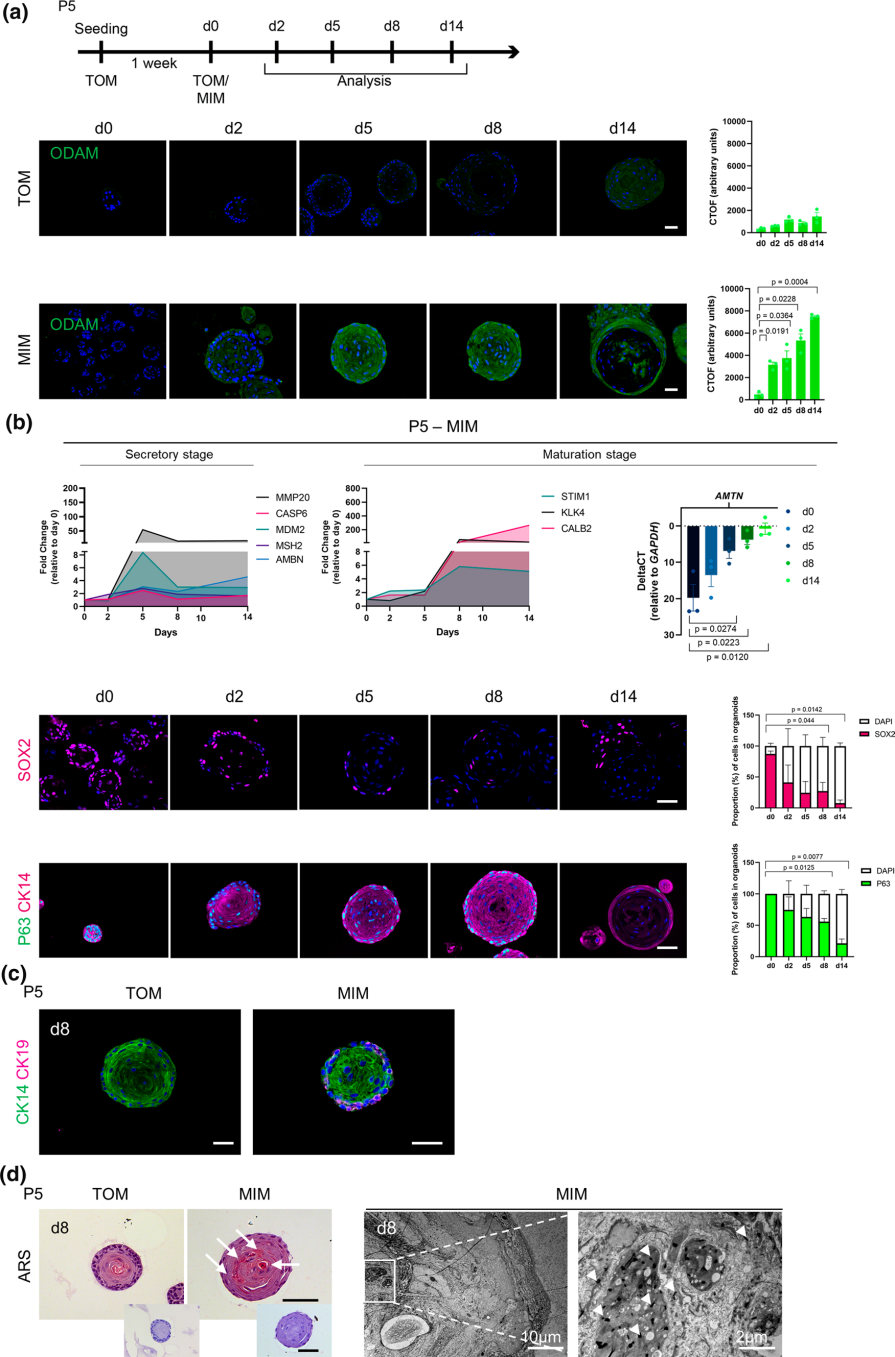
Ameloblast differentiation-mimicking process in tooth organoids. **a** Timeline of experimental set-up (d, day). Immunofluorescence examination of ODAM in organoids from culture conditions and time points as indicated. DAPI (blue) was used to label nuclei. Corrected total organoid fluorescence (CTOF) quantification of ODAM in organoids at indicated time points (mean ± SEM; *n* = 3 biological replicates). **b** Gene expression pattern of ameloblast secretory- and maturation-stage markers in MIM-switched organoids at time points as indicated. Data are expressed as fold change relative to the organoids at switching to MIM (d0). Expression is normalized to expression of *GAPDH*. Data are mean of *n* = 3 biological replicates. Right: Gene expression levels (relative to *GAPDH*) of *AMTN* in MIM-switched organoids at time points as indicated (mean ± SEM; *n* = 3 biological replicates). Below: Immunofluorescence staining for the indicated markers, and quantification of SOX2^+^ and P63^+^ cells in organoids cultured in MIM (mean ± SEM; *n* = 3 biological replicates). DAPI (blue) was used to label nuclei. **c** Immunofluorescence staining for the indicated markers in organoids cultured as specified. DAPI (blue) was used to label nuclei. **d** Alizarin Red S (ARS) staining of organoids cultured as specified. Arrows indicate ARS^+^ areas. Images below show negative control (i.e. hematoxylin only). Right: ultrastructural (TEM) analysis of MIM-switched organoids. Boxed area is enlarged. Arrowheads indicate calcium phosphate crystals. Scale bars: 50 µm, unless indicated otherwise

Organoids expanded in TOM were switched to a medium previously reported to trigger ameloblast-like differentiation in 2D DESC cultures [41, 55, 56] (referred to as mineralization-inducing medium, MIM; Supplementary Table 3), and analyzed at multiple time points (Fig. 4a). Interestingly, ODAM expression swiftly emerged (from day 2) in the organoids switched to MIM, and increased in intensity while remaining absent in TOM-cultured organoids (Fig. 4a). AMELX protein, being already detected in standard TOM conditions (Fig. 1e), became visually more abundant in MIM-switched organoids from day 8, while remaining more constant in TOM-cultured organoids (Supplementary Fig. 4a). Time-lapse gene expression analysis showed that secretory-stage markers of amelogenesis [4, 35, 52, 53] mainly increased at day 5 in MIM, while maturation-stage markers [52, 53] peaked at day 8–14 (Fig. 4b). In contrast, expression did not change in TOM-cultured organoids (Supplementary Fig. 4b). In parallel with the differentiation process, the stemness phenotype of the organoids dropped showing a fast decline in SOX2^+^ and TP63^*+*^ cells (Fig. 4b). Interestingly, CK19 expression emerged in MIM-switched organoids (Fig. 4c), in accordance with the known gradual replacement of CK14 by CK19 in differentiating ameloblasts [57]. Moreover, the MIM-cultured organoids displayed calcium deposits (Fig. 4d; Alizarin red S (ARS) staining), supported by transmission electron microscopy (TEM) revealing the presence of electron-dense calcium-phosphate accumulations (Fig. 4d), analogous to the formation of hydroxyapatite during amelogenesis [53]. This mineralization outcome appeared even more prominent when the MIM-cultured organoids were incubated in an in vivo environment, i.e. following subcutaneous transplantation of 3D-printed hydroxyapatite scaffolds seeded with organoids in immunodeficient mice (Supplementary Fig. 4c; ARS and Masson’s trichrome staining (TCM)).

Finally, to validate whether the ameloblast differentiation capacity is already present in the organoid-initiating ERM stem cells, we developed the organoids immediately in MIM and subsequently analyzed their phenotype (in P1; Supplementary Fig. 4d). ODAM expression was detected in the MIM- (but not TOM-) grown organoids coinciding with the almost absence of SOX2^+^ cells. *AMTN* and *KLK4* were also detected at higher levels in MIM- versus TOM-grown organoids, while *MMP20* expression was only lowly expressed by both organoid types (Supplementary Fig. 4d).

Taken all together, our new tooth organoid model is capable of unfolding an ameloblast differentiation process involving known consecutive steps, thereby recapitulating DESC/ERM functionality, and thus provides a valuable research tool to study amelogenesis of human tooth, at present poorly defined.

### Single-cell transcriptomics of tooth organoids enriches insights into amelogenesis

**Fig. 5:**
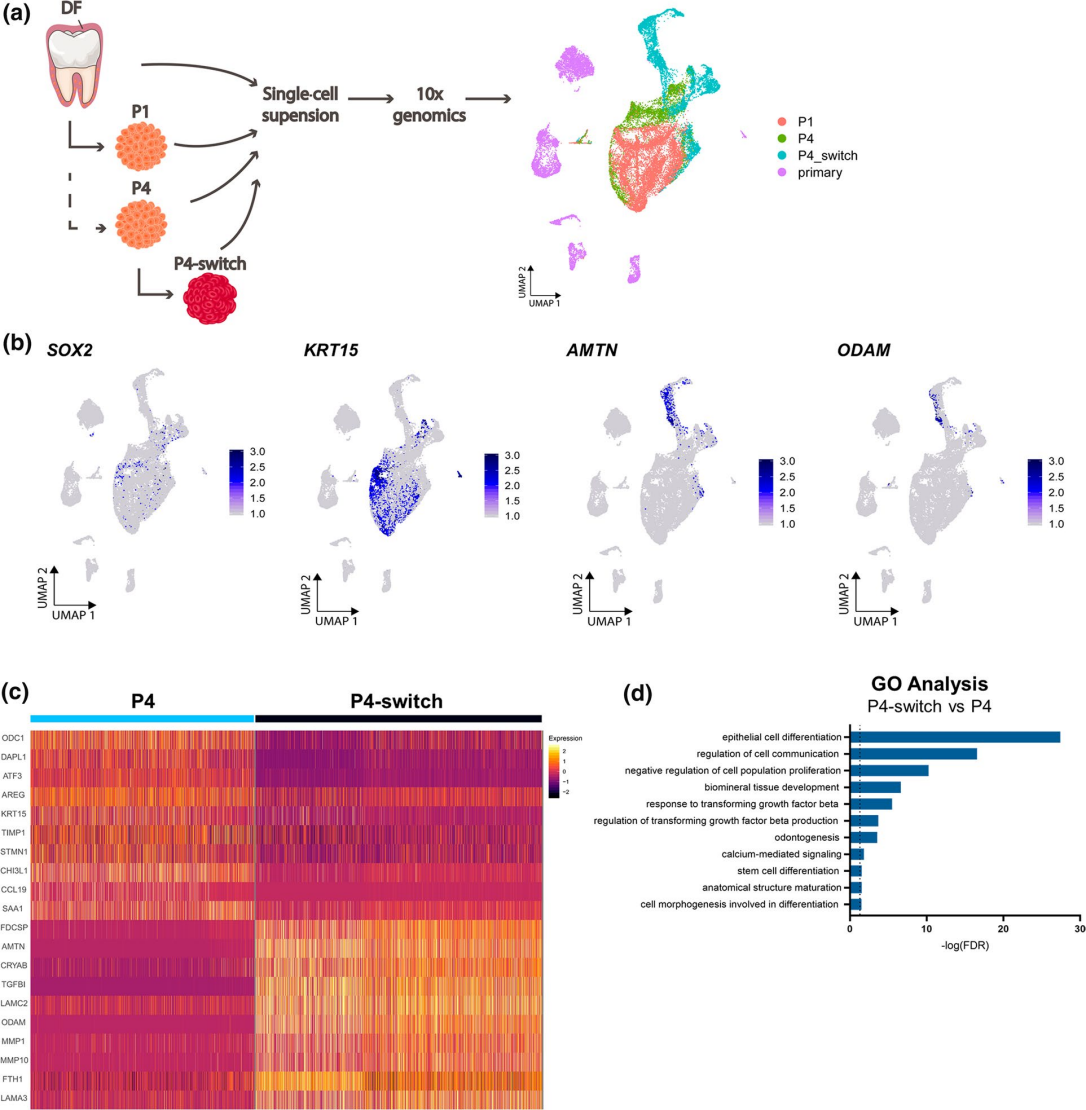

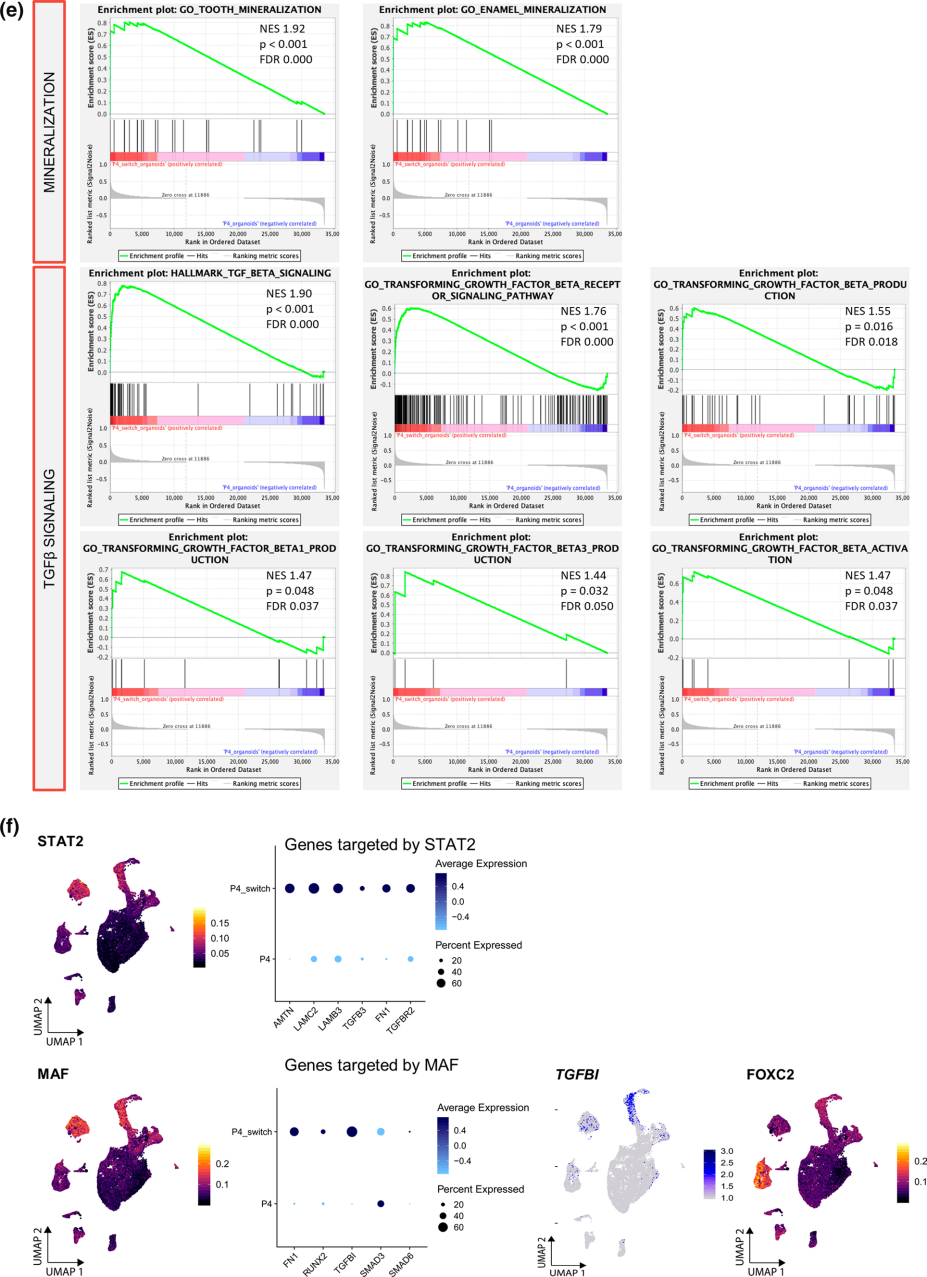
Single-cell transcriptomic profiling of tooth organoids driven into amelogenesis-resembling differentiation. **a** Experimental overview of the scRNA-seq analysis. UMAP plot of the integrated DF and organoid samples as indicated. ‘Primary’ means all DF clusters. **b** Projection of indicated genes on the integrated UMAP plot. **c** Heatmap displaying the scaled expression of the top 10 DEGs per cluster in P4 versus P4-switch organoids. **d** Significant (FDR ≤ 0.05) DEG-based GO terms enriched in P4-switch versus P4 organoids. **e** DEG-based GSEA plots of the indicated hallmarks in P4-switch versus P4 organoids. Normalized enrichment score (NES-), and p- and FDR-values are listed. **f** Indicated regulons (STAT2, MAF, FOXC2) projected on the integrated UMAP plot. Dot plot of predicted STAT2 or MAF regulon target genes in P4 and P4-switch organoids. Projection of *TGFβI *gene expression on the UMAP plot

To decipher the amelogenesis differentiation process that occurs in the tooth organoids in deeper detail, we performed scRNA-seq analysis of P4 organoids switched to MIM for 8 days (referred to as P4-switch; see Fig. 4a), and integrated the data with the scRNA-seq dataset described above (Fig. 5a).

As expected, stemness markers (e.g. *SOX2*, *KRT15*) are more prominent in the non-differentiated P4 organoid cluster, whereas ameloblast differentiation markers (e.g. *AMTN*, *ODAM*) show almost exclusive expression in the differentiated P4-switch organoids (Fig. 5b), all concordant with the findings above. Analogously, the newly identified *EGR1* and *ATF3* are mainly expressed in the non-differentiated P4 organoid cluster, while the tooth development marker *PITX2* was found in both stemness and differentiated organoid groups (Supplementary Fig. 5a).

Looking more broadly at gene expression differences using DEG analysis (Supplementary Dataset 6) revealed that P4 and P4-switch organoid clusters clearly display different gene signatures, thereby exposing interesting (new) markers (Fig. 5c). Among others, ornithine decarboxylase 1 (*ODC1*), a gene involved in cell cycle regulation [58] and proposed as a marker of dental epithelium (moreover aberrantly expressed in specific odontogenic tumors [58]), is higher expressed in P4 versus P4-switch organoids (Fig. 5c). In addition to *KRT15* and *ATF3* belonging to the top 10 upregulated DEGs in P4 organoids (Fig. 5c), other undifferentiated epithelial cell markers are also distinctly expressed in P4 versus P4-switch organoids, including death-associated protein-like 1 (*DAPL1*) and tissue inhibitor of metalloproteinases 1 (*TIMP1*) (Fig. 5c), both recently discovered markers of mouse dental epithelium [46, 59]. On the other hand, in addition to *AMTN* and *ODAM* surfacing in the top 10 DEGs of P4-switch as compared to P4 organoids (Fig. 5c; Supplementary Dataset 6), laminin subunit gamma 2 (*LAMC2*) and laminin subunit alpha 3 (*LAMA3*), both expressed in mature ameloblasts in mouse incisor [46] and essential for amelogenesis in humans (with mutations linked to *amelogenesis imperfecta* [60, 61]), are highly upregulated in P4-switch organoids (Fig. 5c), further validated by RT-qPCR (Supplementary Fig. 5b). Follicular dendritic cell secreted peptide (*FDCSP*), reported to bind to hydroxyapatite [62], is also distinctly expressed in the P4-switch organoids (Fig. 5c).

GO analysis revealed enriched ‘negative regulation of cell differentiation’ in the straight P4 organoids when compared to P4-switch organoids (Supplementary Fig. 5c; Supplementary Dataset 4e, 6). In the reverse comparison, GO analysis exposed enrichment of ‘epithelial cell differentiation’, ‘biomineral tissue development’, ‘odontogenesis’ and ‘calcium-mediated signaling’ in P4-switch versus P4 organoids (Fig. 5d; Supplementary Dataset 4f). Interestingly, also TGFβ-associated processes are upregulated (Fig. 5d), in line with the enrichment of ‘negative regulation of SMAD protein signal transduction’ in the non-differentiated organoids (Supplementary Fig. 5c), and the knowledge that the TGFβ pathway plays an important role in ameloblast differentiation [63].

Next, we performed gene set enrichment analysis (GSEA) [64] which exposed several important differentiation (amelogenesis) pathways in P4-switch versus P4 organoids. Firstly, mineralization hallmarks (tooth, enamel) are significantly enriched in P4-switch organoids (Fig. 5e). In addition, calcium-signaling pathways, highly important during amelogenesis [53], were found significantly associated with the P4-switch organoids such as the hallmarks ‘calmodulin binding’, ‘store-operated calcium entry’ and ‘calcium mediated signaling’ (Supplementary Fig. 5d). Further interestingly, GSEA revealed significant enrichment of TGFβ signaling hallmarks in P4-switch versus P4 organoids, more specifically TGFβ (receptor) signaling and TGFβ (particularly TGFβ1/3) production (Fig. 5e), in line with the importance of the TGFβ pathway in amelogenesis [63].

Regulon analysis exposed higher activity of the signal transducer and activator of transcription 2 (STAT2) gene-regulatory network in P4-switch than P4 organoids (Fig. 5f). STAT2 is specifically found in ameloblasts (reported in neonatal rat molars [65]) and positively targets *AMTN*, the ameloblast-related *LAMC2* and *LAMB3*, as well as genes associated with TGFβ signaling (*TGFβ3*, *TGFβR2*) (Fig. 5f). Also, avian musculoaponeurotic fibrosarcoma (MAF), specifically expressed in ameloblasts (reported in mouse incisor tooth germs [66]) and representing an essential regulator of AMELX secretion during amelogenesis [66], shows higher regulon activity in P4-switch than P4 organoids (Fig. 5f). MAF is predicted to positively regulate fibronectin (*FN1*) and *RUNX2*, genes related to ameloblast differentiation, and TGFβ signaling-associated *SMAD3*, *SMAD6*, and TGFβ-induced (*TGFβI*), an activated form of the TGFβ1 ligand (Fig. 5f). RUNX2 expression has been reported in ameloblasts during the late secretory and maturation stages and its deletion suppresses enamel maturation [67]. In addition, TGFβ1 affects enamel mineralization by modulating RUNX2 [67, 68]. *TGFβI* is also an important predicted target gene of Forkhead Box C2 (FOXC2), and FOXC2 regulon activity was found higher in P4-switch than P4 organoids (Fig. 5f). FOXC2 is highly expressed during craniofacial development [69], but its exact role during tooth development and differentiation is unknown. FOXC2 is also predicted to positively regulate *LAMC2* and *MSX1*, a highly conserved transcription factor well-known to regulate tooth formation [4], and causing tooth agenesis in humans when mutated [70]. Finally, SOX4 and HMGA2 regulons are prominently activated in P4-switch organoids (Supplementary Fig. 5e). SOX4 expression has been reported in DESCs and in inner enamel epithelium (at the cap stage in mouse) [71] and targets *PITX2*, while HMGA2 is involved in early tooth formation and stem cell marker (e.g. SOX2) expression [72] (hence, plausibly associated with enlarged and supernumerary teeth when truncated [72]), and predicted targets include *FN1* and *LAMA3* (Supplementary Fig. 5e).

Pseudotime trajectory analysis (using Monocle3 [73]) projected a potential developmental path from P1-P4 to P4-switch clusters (Supplementary Fig. 5f). Intriguingly, the trajectory passes through a particular subcluster of the P4-switch organoids (Supplementary Fig. 5f, encircled), likely representing a transitional stage as supported by the concurrent expression of stemness/development markers (*SOX2*, *KRT15*, *PITX2*) and differentiation markers (*AMTN*, *ODAM*) in this subcluster (see Fig. 5b; Supplementary Fig. 5a). Interestingly, regulons controlling ameloblast differentiation (PITX1, DLX3, MEIS1) are especially active in this subcluster (Supplementary Fig. 5g). PITX1 is required for proper tooth formation [74], and has been described in secretory stage ameloblasts [75]. DLX3 promotes the expression of EMPs during amelogenesis [76], and MEIS1 has been shown to bind to DLX3 [77].

In a final analysis of the scRNA-seq data, we applied STRING to in silico predict protein–protein interactions [78]. Using the top 40 DEGs in P4-switch versus P4 organoid clusters, it is projected that AMTN and ODAM closely interact (Supplementary Fig. 5h; Supplementary Dataset 6, 7a), in agreement with former reports [76]. Interestingly, AMTN is also predicted to cooperate with C4orf26, an ECM acidic phosphoprotein suggested to play a key role in enamel mineralization and crystal nucleation [76]. In addition, the STRING analysis proposed interaction of AMTN with LAMB3 suggesting a role in cell–matrix attachment, in line with previously proposed interactions of AMTN with laminins localized in the epithelial basement membrane of the ECM [76]. In the predicted network, LAMB3 interacts with LAMA3 and LAMC2 which is consistent with previous reports [76]. Interestingly, AMTN is also proposed to network with FN1, at present not reported. Moreover, FN1 is predicted to interact with TGFβ1 and ITGβ6. ITGβ6 is known to activate TGFβ1 by binding to arginine-glycine-aspartic acid (RGD) motifs present in ECM proteins such as FN1 [76]. GO-Biological Process analysis of these particular top 40 DEGs in P4-switch organoids confirmed key features of biomineral tissue development, odontogenesis and enamel mineralization, as well as of TGFβ signaling, the latter further stressed by KEGG pathway analysis (Supplementary Fig. 5i; Supplementary Dataset 7b,c).

Taken together, single-cell transcriptomics of the tooth organoids driven into amelogenesis differentiation demonstrates and underscores the relevance of our new organoid model by confirming known data as well as presenting new insights in the amelogenesis process in humans which is at present far from clarified.

### TGFβ fortifies amelogenesis in tooth organoids and triggers PDL-like differentiation

**Fig. 6:**
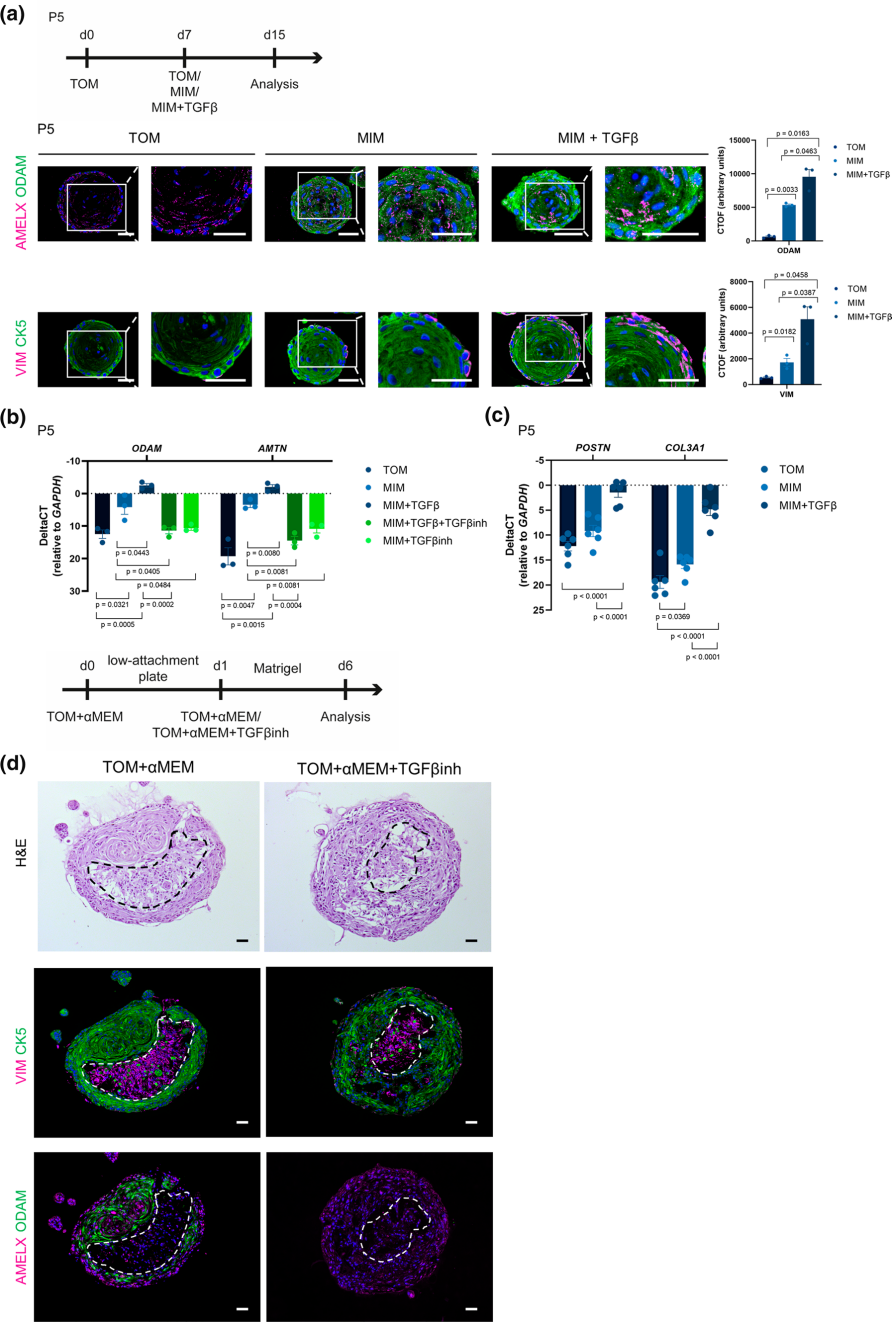
Effect of TGFβ on differentiation in tooth organoids. **a** Timeline of experimental set-up (d, day). Immunofluorescence examination for the indicated markers in organoids cultured as denoted. Boxed areas are enlarged. DAPI (blue) was used to label the nuclei. CTOF quantification of indicated markers in organoids cultured as specified (mean ± SEM; *n* = 3 biological replicates). **b-c** Gene expression levels (relative to *GAPDH*) of indicated markers in organoids cultured as denoted (mean ± SEM; *n* ≥ 3 biological replicates). **d** Timeline of experimental set-up. Histological (H&E) analysis and immunofluorescence examination for the indicated markers in assembloids cultured as indicated. DAPI (blue) was used to label the nuclei. Dotted area demarcates the (VIM^+^) mesenchymal cells. Scale bars: 50 µm

The above analyses exposed the enrichment of TGFβ pathway processes in tooth organoids subjected to ameloblast differentiation, in line with the proposed key role of TGFβ in amelogenesis [63]. To assess the impact of the TGFβ pathway, we switched organoids grown in TOM (P5) to MIM with or without TGFβ (Fig. 6a). Immunofluorescence analysis revealed that addition of TGFβ further upregulated the expression of ODAM (Fig. 6a), supported by gene expression interrogation also showing increased expression of *AMTN* (Fig. 6b). The effect of TGFβ was blocked by the simultaneous addition of a TGFβ receptor inhibitor (LY2109761, further referred to as TGFβinh), thereby demonstrating the specificity of the effect observed (Fig. 6b). Intriguingly, adding TGFβinh to MIM-cultured organoids (i.e. without adding exogenous TGFβ) strongly reduced the upregulated *ODAM* and *AMTN* expression in MIM (Fig. 6b), indicating the presence and implication of endogenous TGFβ signaling in the differentiation process, in line with our findings above (Fig. 5d,e) and corroborated by the increase in expression of *TGFβ1* and its receptors *TGFβR1* and *TGFβR2* in MIM culture (Supplementary Fig. 6a). Together, the data demonstrate that TGFβ further advances the amelogenesis-mimicking differentiation in the tooth organoids.

During tooth root development, Hertwig’s epithelial root sheath (HERS), from which ERM is eventually derived [4, 5], undergoes EMT to develop to participate in PDL formation [47]. It has previously been proposed that this EMT process is regulated by TGFβ, thereby triggering HERS/ERM cells to switch phenotype toward PDL cells [5, 47, 79]. Addition of TGFβ indeed further increased the expression of VIM in the epithelial (CK5^+^) organoids (Fig. 6a) and also significantly stimulated the expression of the PDL-specific genes periostin (*POSTN*) and collagen type III alpha 1 chain (*COL3A1*) [80] (Fig. 6c).

Taken together, TGFβ coerces the tooth organoids into more pronounced ameloblast differentiation as well as into the direction of PDL development. These findings conform to the known activity of TGFβ in these tooth developmental processes, and thus again corroborate the strength and validity of our new organoid model. Moreover, they provide supportive evidence that the DF-derived organoids replicate the multipotency of dental (HERS/ERM) epithelial stem cells as proposed to unfold in vivo during tooth development and possibly repair [5, 26].

### The presence of tooth mesenchymal cells triggers ameloblast differentiation in the epithelial organoids

Given the importance of mesenchyme-epithelium interactions during tooth development including ameloblast differentiation/amelogenesis [4, 54], we addressed the question whether addition of dental mesenchymal cells had an impact on ameloblast differentiation of the epithelial organoids. We opted to use DPSCs to mimic early stages of tooth development in which DPSC-derived odontoblasts are in close contact with ameloblasts [4]. The DPSCs, isolated, grown and characterized using well-defined standard protocols [81–83], were combined with organoid-derived epithelial stem cells in a layered approach [20], thereby forming composite organoids (assembloids) which were cultured in a mixture of TOM and the DPSC growth medium αMEM (Fig. 6d). The hybrid epithelial-mesenchymal composition was confirmed by CK5-VIM immunofluorescence analysis (Fig. 6d), revealing VIM^+^ mesenchymal cells in the inner part and CK5^+^ epithelial cells at the outer zone of the assembloids (Fig. 6d), and by developing assembloids using eGFP-expressing DPSCs (Supplementary Fig. 6b).

Whereas ODAM is not present in the straight (pure) epithelial organoids cultured in TOM (see above and Supplementary Fig. 6c), it is expressed in the assembloids (Fig. 6d). This induction is not due to the addition of αMEM to TOM (Supplementary Fig. 6c). Interestingly, the epithelial cells neighboring the DPSCs express ODAM, whereas the cells at the outside border of the assembloids (thus, not in direct contact with the mesenchymal cells while more exposed to the (stem cell) medium) do not (Fig. 6d). These findings indicate that the presence of (and even more, the close interface with) mesenchymal (stem) cells drives epithelial stem cells into ameloblast differentiation. Addition of TGFβinh completely abolished ODAM protein expression in the assembloids (Fig. 6d) and reduced the expression of *AMTN* (Supplementary Fig. 6d), indicating the involvement of endogenous TGFβ signaling in the observed effects. TGFβ pathway components are indeed expressed in the assembloid culture (Supplementary Fig. 6e); the ligand(s) may originate from the epithelial cells (see Supplementary Fig. 6a), further upregulated by the presence of mesenchymal cells, or may be additionally produced by the mesenchymal cells since both dental cell types have been shown to produce TGFβ [84, 85].

Taken together, ameloblast differentiation of epithelial (organoid) stem cells is triggered by the presence of tooth mesenchymal cells involving TGFβ signaling, thereby corroborating in vivo findings of interactive mesenchyme-epithelium importance, and further validating our model as valuable research tool for exploring human tooth (stem cell) biology.

## Discussion

To our knowledge, our study reports the first-in-time development of a long-term expandable epithelial organoid model derived from human dental tissue. The DF-derived organoids show a stemness expression profile congruent with the ERM, previously advanced to encompass DESCs [5]. In addition, single-cell transcriptomics uncovered novel molecular features (such as the stemness-associated hybrid E/M nature, new markers and gene-regulatory networks) for the as yet ill-defined and poorly comprehended human DESCs and ERM, interestingly often mirroring very recent findings in mouse [43, 46]. Noticeably, organoid culturing appeared to proliferatively (re-)activate the ERM stem cells, indeed reported to be highly quiescent in vivo [7, 12]. Moreover, described (stem cell-related) functional properties of the ERM were markedly recapitulated by the tooth organoids. First, exposure to EGF induced transient proliferation and eventual EMT and migration, thereby mimicking events taking place in the ERM in vivo (for instance, upon tooth insult) [5, 26, 51]. Second, the tooth organoids displayed the capacity to unfold an ameloblast differentiation process, as occurring in vivo during tooth formation [4] and reported for ERM [6, 7, 35, 54], thus recapitulating this ERM differentiation capacity. The organoids displayed molecular changes constituting pathways that underlie ameloblast differentiation during amelogenesis [52, 53]. In addition, the organoids recovered the key position of TGFβ in ameloblast differentiation/amelogenesis [63, 67, 76], as well as in PDL development [5, 47, 79, 80]. Moreover, our scRNA-seq interrogation advanced molecular transitions not revealed before in human amelogenesis. Also, STRING analysis projected protein–protein interactions that may further deepen our knowledge on amelogenesis in human tooth, at present not understood. Together, our new model has the potential to in detail decipher ameloblast development and their production of enamel, the quintessential component of our teeth, which would represent an enormous leap forward in the dental field (especially for future dental tissue replacement therapies). Third, the organoid transcriptome reflected functional processes before (provisionally) assigned to the ERM [5, 26], including regulation of bone mineralization, osteoblast differentiation and tooth eruption. Hence, our new model may also serve as an interesting tool to help decipher the multiple biological functions assigned to the ERM, at present still debated [5]. Importantly, the organoids show strong expandability, thereby overcoming current hurdles of primary ERM/DESC culturing, such as limited cell number, life span and phenotypical loss. The expansion ability will be highly instrumental for allowing in-depth analysis of this yet enigmatic cell population. Finally, the induction of ameloblast differentiation by the presence of mesenchymal cells, thereby recapitulating the acknowledged importance of epithelium-mesenchyme interaction in tooth development including amelogenesis, again further corroborated the biomimetic value of our new model(s). Altogether, the several characterizations provide strong evidence that our new human tooth (DF)-derived organoid models, to our knowledge not developed before, present a valuable tool to study human tooth epithelial stem-cell biology and development, at present far from understood.

Organoid technology is also highly applicable to human disease modeling in vitro. It has been suggested that ERM cells are associated with the pathogenesis of odontogenic cysts and tumors [5]. Developing organoids from these lesions may help to gain better insight in their pathogenesis. More in general, our tooth organoid approach may be harnessed to model and study tooth diseases ranging from impact of bacteria to genetic mutations (like mutations in *P63* and *PITX2* [33] associated with tooth anomalies and *amelogenesis imperfect* [60, 61]), eventually leading to novel therapeutic targets and treatments.

Finally, organoids have been shown amenable to regenerative replacement therapy [86]. It is tempting to speculate that damaged, lost or missing teeth, causing major health problems [1–3], may in the future be regenerated or replaced by transplanting biological tooth constructs. Such approach may be superior (both material- and function-wise) to the traditional, still suboptimal synthetic implants, among others suffering from lack of physiological functionality, inferior bone integration and absence of innervation. Embryonically derived, bioengineered mouse tooth germs have been shown capable of forming a functional tooth unit after transplantation in an emptied dental cavity of the mouse [18, 20]. Our organoid and assembloid models may provide essential puzzle pieces toward developing human tooth germs. Although transplantation of natural teeth has been performed in some patients, especially children and young adolescents, the availability of such teeth remains limited. Of important note, the murine Matrigel should then be replaced by a clinically compatible ECM mimic. Currently, attempts are being made to substitute Matrigel for defined synthetic hydrogels [87, 88], although achievements are still limited. In conclusion, we developed a long-term expandable stemness organoid model from human tooth, replicating molecular and functional features of the originating epithelial stem cell compartment. The new in vitro model will be highly valuable to explore human tooth epithelial stem cell phenotype and biology such as ameloblast differentiation. Moreover, our study indicates that the postnatal human tooth still contains epithelial stem cells, and the organoids will be beneficial to address the question on their role(s), and on the reasons why they do not, or not prominently, regenerate tooth tissue in postnatal life. This search also implicates the question whether these stem cells can in vivo be re-activated for repair. This understanding may eventually instigate tooth-regenerative approaches by re-activating endogenous repair capacity and processes.

## Material & methods

### Isolation and dissociation of dental follicle

Third molars, predominantly unerupted, were extracted from adolescent patients (Supplementary Table 2) at the ‘Oral and Maxillo-Facial Surgery—Imaging & Pathology (OMFS-IMPATH)’ unit of University Hospitals (UZ) Leuven after informed consent. The study was approved by the Ethics Committee Research UZ/KU Leuven (13/0104U). For sample collection, the gingiva was pushed aside after which the bone was perforated and the third molars with associated DFs were carefully isolated (without the visually distinct gingiva). DF tissue was diligently peeled from the tooth and collected in Eagle’s Minimum Essential Medium (αMEM; Sigma-Aldrich) supplemented with 10% fetal bovine serum (FBS; Sigma-Aldrich), 1% penicillin–streptomycin (Gibco) and 0.5% fungizone (Amphotericin B; Gibco). Following short rinsing steps in 70% ethanol and phosphate-buffered saline (PBS; Gibco), tissue was minced into small (~ 1 mm^2^) fragments, and further dissociated using collagenase VI (3 mg/ml; Thermo Fisher Scientific) and dispase II (4 mg/ml; Sigma-Aldrich) for 2 h at 37 °C, while regularly pipetting up and down. The single cells and few small cell clusters were collected through a 40 µm cell strainer (Corning) while removing the remaining larger and fibrous tissue fragments.

### Establishment and passaging of tooth organoid culture

The dissociated DF cell material was resuspended in a mixture of serum-free defined medium (SFDM; Thermo Fisher Scientific; Supplementary Table 4 and [89]) and growth factor-reduced Matrigel (Corning) in a 30:70 ratio, which was plated in 48-well plates at 20,000 cells per 20 µL drop. After solidification, tooth organoid medium (TOM; Supplementary Table 1), unless indicated otherwise, was supplemented. ROCK inhibitor (RI; 10 µM; Merck Millipore) was added the first day of seeding (or passaging). Organoid cultures were kept at 37 °C in a 1.9% CO_2_ incubator, and medium was refreshed every 2–3 days, each time supplemented with fungizone (0.1%).

The organoid cultures were passaged every 10–14 days. Matrigel droplets were collected using ice-cold SFDM, and organoids dissociated using TrypLE (containing 5 µM RI; Thermo Fisher Scientific) and mechanical trituration. Remaining large organoid fragments were allowed to sediment and the supernatant, containing single cells and small fragments, seeded as described above. A split ratio of 1:6 was applied once the culture reached stable growth (typically from P2 to P4). Organoids were cryopreserved as previously described [14, 15] and stored in liquid nitrogen.

To assess clonal derivation, dissociated single organoid cells were transduced with the lentiviral vector LV-eGFP [90] during 30 min at 37 °C, resulting in 60% eGFP^+^ cells as analyzed by flow cytometry. The resulting mixture of eGFP^+^ and eGFP^−^ cells was seeded in organoid culture as described above, and cultures analyzed 14 days later using brightfield and epifluorescence microscopy (Axiovert 40 CFL; Zeiss).

### FACS isolation of ITGα6^±^ cells from DF

Primary DFs were dissociated into single cells as described above. Cells were incubated with PE-anti-ITGα6 antibody (1:5; Cat.no 555736; BD Biosciences) and rinsed, both performed in TOM supplemented with fungizone (0.1%) and RI (10 µM). ITGα6^+^ and ITGα6^−^ cells within the living (DAPI-negative) population were sorted in TOM (supplemented with fungizone and RI) using a BD Influx (BD Biosciences), and seeded at 7500 cells per 20 µL Matrigel droplet as mentioned above. RI (10 µM) was added to the cultures for 1 week.

### In vitro differentiation of the DF-derived epithelial organoids

Organoids (or dissociated DF) were cultured in mineralization-inducing medium (MIM; Supplementary Table 3; time schedule, see Fig. 4a and Supplementary Fig. 4d) as described above. Recombinant human TGFβ1 (10 ng/ml; R&D) and the selective TGFβ receptor 1/2 inhibitor LY2109761 (5 µM; Selleckchem) were added when indicated.

### In vivo transplantation of the DF-derived epithelial organoids

Matrigel (10 µL) with dissociated organoid cells (150,000) was pipetted into custom-made 3D-printed hydroxyapatite constructs (Sirris) which were subcutaneously transplanted in immunodeficient nu/nu mice (Janvier Labs), as in detail described elsewhere [91]. After 4 weeks, implants were resected and subjected to 48 h-fixation in 4% paraformaldehyde (PFA) (Sigma-Aldrich), paraffin-embedding, 24 h-decalcification, 7-µm sectioning and Alizarin Red S (ARS) or Masson’s Trichrome (TCM) staining as described [91]. The study was approved by the Ethical Committee on Animal Experiments (ECAE) of Hasselt University (protocol 202,044).

### Dental pulp stem cell culture

DPSCs were obtained as in detail described and characterized before [81–83]. In short, dental pulp was collected from the extracted wisdom teeth (after careful removal of the apical papilla), minced and fragments cultured in T25 flasks (Corning) in αMEM supplemented with 10% fetal bovine serum (FBS) and 1% L-glutamine (Gibco). When 70–80% confluence was reached, cells were trypsinized and re-plated at 150,000 cells per T75 flask, and used at early passage (~ P5) for assembloid creation. For GFP labelling, DPSCs were transduced with the lentiviral vector LV-eGFP [90] as described above.

### Development and culture of assembloids

Organoid and DPSC cultures were dissociated into single cells, and mixed in a round-bottom low-attachment plate (96-well; Greiner) using a layered approach [20]. First, DPSCs (5 × 10^4^ cells) were sedimented by centrifugation (300 g for 1 min at 4 °C), followed by deposition of the organoid-derived cells (1 × 10^5^; at 300 g and 4 °C for 2 min). The cells were layered in 10% Matrigel and 90% of a 1:1 mixture of TOM (i.e. organoid growth medium) and αMEM (i.e. DPSC growth medium), and then incubated for 24 h at 37 °C in 5% CO_2_. The formed aggregate was re-plated into a 48-well plate in a 20 µL Matrigel (70%) droplet as described above to generate the assembloid, further cultured in TOM + αMEM with or without the TGFβ receptor inhibitor LY2109761 (5 µM) as indicated.

### Histochemical and immunostaining analysis

Primary DF tissue, organoids and assembloids were fixed in 4% PFA for 1 h, embedded in paraffin, and sections subjected to hematoxylin and eosin (H&E), immunofluorescence or ARS staining. Antigen retrieval (10 mM citrate, pH6) and permeabilization (0.1% Triton X-100; Sigma-Aldrich) were performed. After incubation with primary and secondary antibodies (Supplementary Table 5), sections were mounted with Vectashield (DAPI; Vector Laboratories) or DPX mountant (Sigma-Aldrich). Analysis was done using a Leica DM5500 epifluorescence microscope or a Zeiss Axioimager epifluorescence microscope. ImageJ software was used to quantify immunoreactive signal intensity and the ‘corrected total organoid fluorescence’ (CTOF) (= integrated density—(area of organoid × mean fluorescence of background readings).

### Transmission electron microscopy

Organoid samples were prepared for transmission electron microscopy (TEM) as in detail described before [15, 50]. In short, samples were fixed in glutaraldehyde/osmium tetroxide, dehydrated, embedded in epoxy resin, and cut into 40–70 nm sections. TEM analysis was performed with the JEM1400 transmission electron microscope (JEOL) equipped with an Olympus SIS Quesmesa 11 Mpxl camera, or the Philips EM208 S electron microscope (Philips) equipped with the Morada Soft Imaging System camera with corresponding iTEM-FEI software (Olympus SIS).

### Gene expression analysis by RT-qPCR

RNA was extracted from dissociated DF, organoids and assembloids using the GenElute Mammalian Total RNA Miniprep Kit (Sigma-Aldrich) according to the manufacturer’s instructions. RNA was reverse-transcribed (RT) using the Superscript III First-Strand Synthesis Supermix (Thermo Fisher Scientific) and the resultant cDNA samples were analyzed with SYBR Green-based quantitative PCR (qPCR) using the StepOnePlus Real-Time PCR System (AB applied biosystems). Forward and reverse primers (Supplementary Table 6) were designed using PrimerBank and PrimerBlast. Glyceraldehyde-3-phosphate dehydrogenase (*GAPDH*) was included as housekeeping gene. Relative gene expression levels were calculated as ΔCt (Ct target—Ct housekeeping gene) and compared to control (see figure legends).

### Single-cell RNA-sequencing analysis

Primary DF tissue from two individual patients (see Supplementary Table 2), and derived organoids at P1 and P4, or switched to MIM (P4-switch), were dissociated into single cells (as described above) and subjected to scRNA-seq analyses using 10 × Genomics, according to manufacturer instructions. Libraries were generated using the Chromium Single Cell 3′ v2 Chemistry Library Kit, Gel Bead & Multiplex kit (10 × Genomics), and sequenced on NovaSeq6000. After quality control, raw sequencing reads were demultiplexed, aligned to the human reference genome GRCh38 and processed to a matrix representing the UMI’s per cell barcode per gene using CellRanger (v3; 10 × Genomics). Downstream analysis was performed in R (v.3.6.1) using Seurat (v.3.0) [92].

First, data from the primary DF tissue, P1 and P4 organoids were integrated and analyzed, and subsequently data from P4-switch organoids were added for a next analysis (further referred to as Integration 1 and Integration 2, respectively). Dead cells and potential doublets (i.e. with < 300 genes or  > 10,000 genes,  > 150,000 unique molecular identifiers (UMI) and  > 15% mitochondrial RNA) were removed (Supplementary Fig. 2b), resulting in a total of 22,317 cells in Integration 1 and 27,851 cells in Integration 2. Integration anchors were identified using the FindIntegrationAnchors function with default parameters and dims = 1:30, and data were integrated across all features. Next, expression levels were scaled, centered and subjected to principal component analysis (PCA). The top 30 PCs were selected and used for UMAP dimensionality reduction [42]. Clusters were calculated by the FindClusters function with a resolution set to 2 and 0.1 for Integration 1 and Integration 2, respectively. Differential gene expression was calculated for each cluster using the MAST package (v.1.12.0; Supplementary Dataset 1). All clusters were annotated based on reported DF and ERM markers and on recent scRNA-seq studies of mouse teeth [12, 27, 28, 33, 34, 43, 46]. Marker genes were defined using FindAllMarkers in Seurat.

Gene ontology analysis (GO) of biological processes was done in Panther [44] using significant differentially expressed genes (DEGs; FDR ≤ 0.05 and logFC ≥ 0.25). Gene-regulatory networks (regulons) were identified using SCENIC (pySCENIC; v.0.9.15) [45] in Python (v.3.6.9) as described before [89]. In short, co-expression modules were generated and regulons inferred (with default parameters and hg38__refseq-r80__10kb_up_and_down_tss.mc9nr.feather and hg38__refseq-r80__500bp_up_and_100bp_down_tss.mc9nr.feather motif collections) resulting in a matrix of AUCell values that represent the activity of each regulon in each cell. The AUCell matrix was imported into Seurat and regulons were projected on the integrated UMAP plot.

Gene-set enrichment analysis (GSEA; v.4.1.0) [64, 89] was performed on P4 and P4-switch organoids using normalized expression data. Gene sets (hallmarks) tested were obtained from the Molecular Signatures Database (MSigDB; v.7.2) [64, 93].

To predict protein–protein interactions with STRING (v.11.0) [78], the top 40 DEGs of P4-switch organoids versus P4 organoids were used. The cluster analysis was subdivided in three colors by kmeans. The minimum required interaction score was set as medium confidence (0.4).

Finally, the pseudotime trajectory was projected onto the integrated UMAP dimensional reduction generated previously with Seurat (P1, P4 and P4-switch organoids) using the Monocle3 (v1.0.0) [73] package’s learn_graph and plot_cells functions.

Raw sequencing data are available at ArrayExpress (accession number E-MTAB-10596).

### Statistical analysis

Statistical analysis was performed using GraphPad Prism (v.9.0.0). (Un-)paired two-tailed *t*-student test was applied for comparison of 2 groups or two-way analysis of variance (ANOVA) for multiple comparisons followed by Sidak’s test for Multiple Comparison. Statistical significance was defined as *P* ≤ 0.05.

## Supplementary Information

Below is the link to the electronic supplementary material.

Supplementary file1 (XLSX 473 KB)

Supplementary file2 (XLSX 147 KB)

Supplementary file3 (XLSX 147 KB)

Supplementary file4 (XLSX 377 KB)

Supplementary file5 (XLSX 153 KB)

Supplementary file6 (XLSX 151 KB)

Supplementary file7 (XLSX 29 KB)

Supplementary file7 (PDF 1771 KB)

## Data Availability

The data that support the findings discussed here are available from the corresponding author upon reasonable request.
