# Supplementary material for: Organoids from human tooth showing epithelial stemness phenotype and differentiation potential
Source: Cell Mol Life Sci. 2022 Feb 26;79(3):153. doi: 10.1007/s00018-022-04183-8 (PMC8881251; doi:10.1007/s00018-022-04183-8)
Supplement: Supplementary file 8 — Supplementary file7 (PDF 1771 KB) [file 18_2022_4183_MOESM8_ESM.pdf]

Organoids from human tooth showing epithelial stemness phenotype and differentiation potential

Cellular and Molecular Life Sciences

Lara Hemeryck[1], Florian Hermans[1,2], Joel Chappell[3], Hiroto Kobayashi[4], Diether Lambrechts[5,6], Ivo Lambrichts[2], Annelies Bronckaers[2], Hugo Vankelecom[1*]

[1]Laboratory of Tissue Plasticity in Health and Disease, Cluster of Stem Cell and Developmental Biology, Department of Development and Regeneration, Leuven Stem Cell Institute, KU Leuven (University of Leuven), Leuven, Belgium

[2]Faculty of Medicine and Life Sciences, Biomedical Research Institute (BIOMED), UHasselt (Hasselt University), Diepenbeek, Belgium

[3]Bit.bio, Department of Bioinformatics, Babraham Research Campus, Cambridge, United Kingdom

[4]Department of Anatomy and Structural Science, Yamagata University Faculty of Medicine, Yamagata, Japan

[5]Center for Cancer Biology, VIB, Leuven, Belgium

[6]Laboratory for Translational Genetics, Department of Human Genetics, KU Leuven, Leuven, Belgium

*Correspondence should be addressed to H.V. (hugo.vankelecom@kuleuven.be)

SUPPLEMENTARY FIGURES

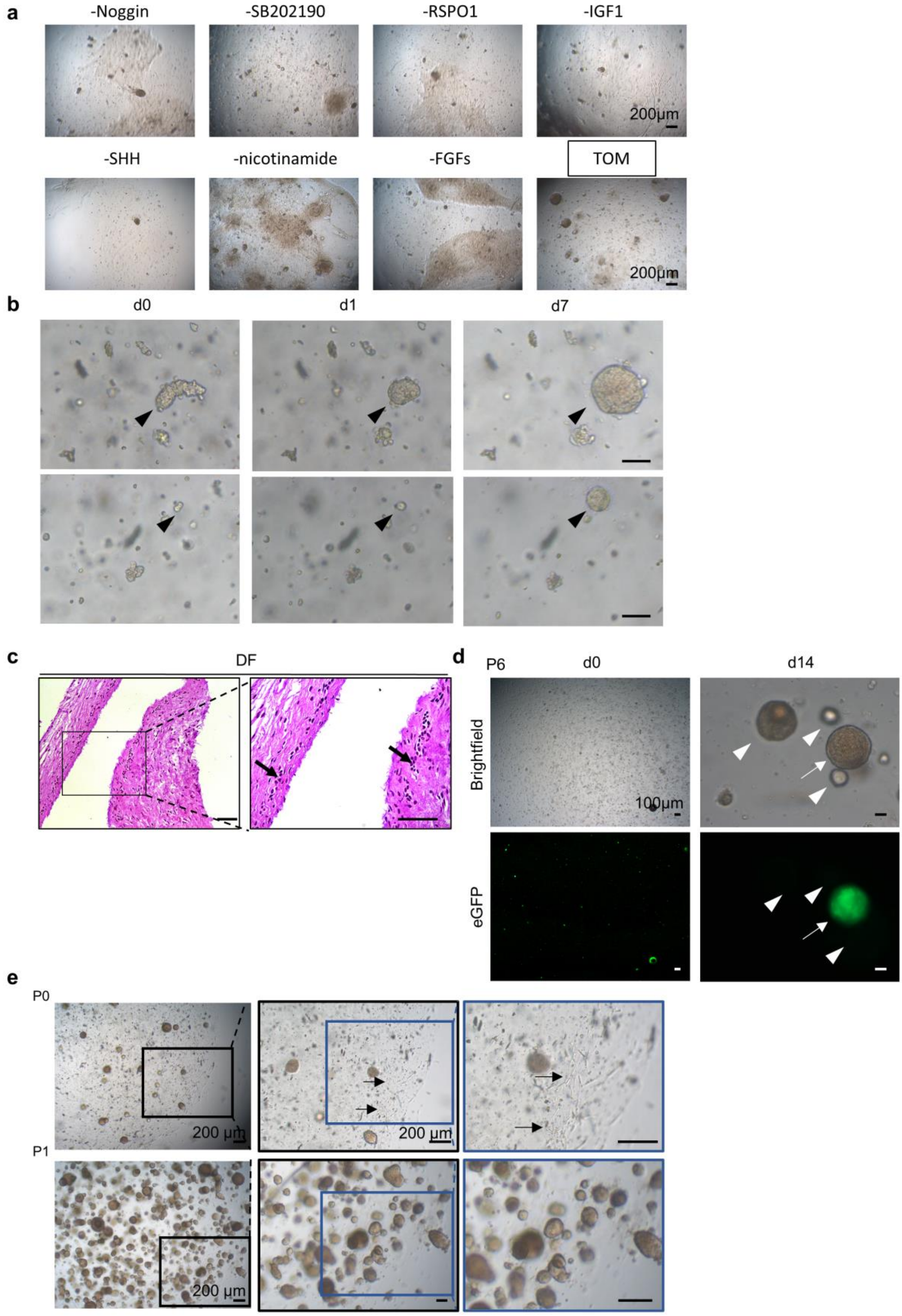

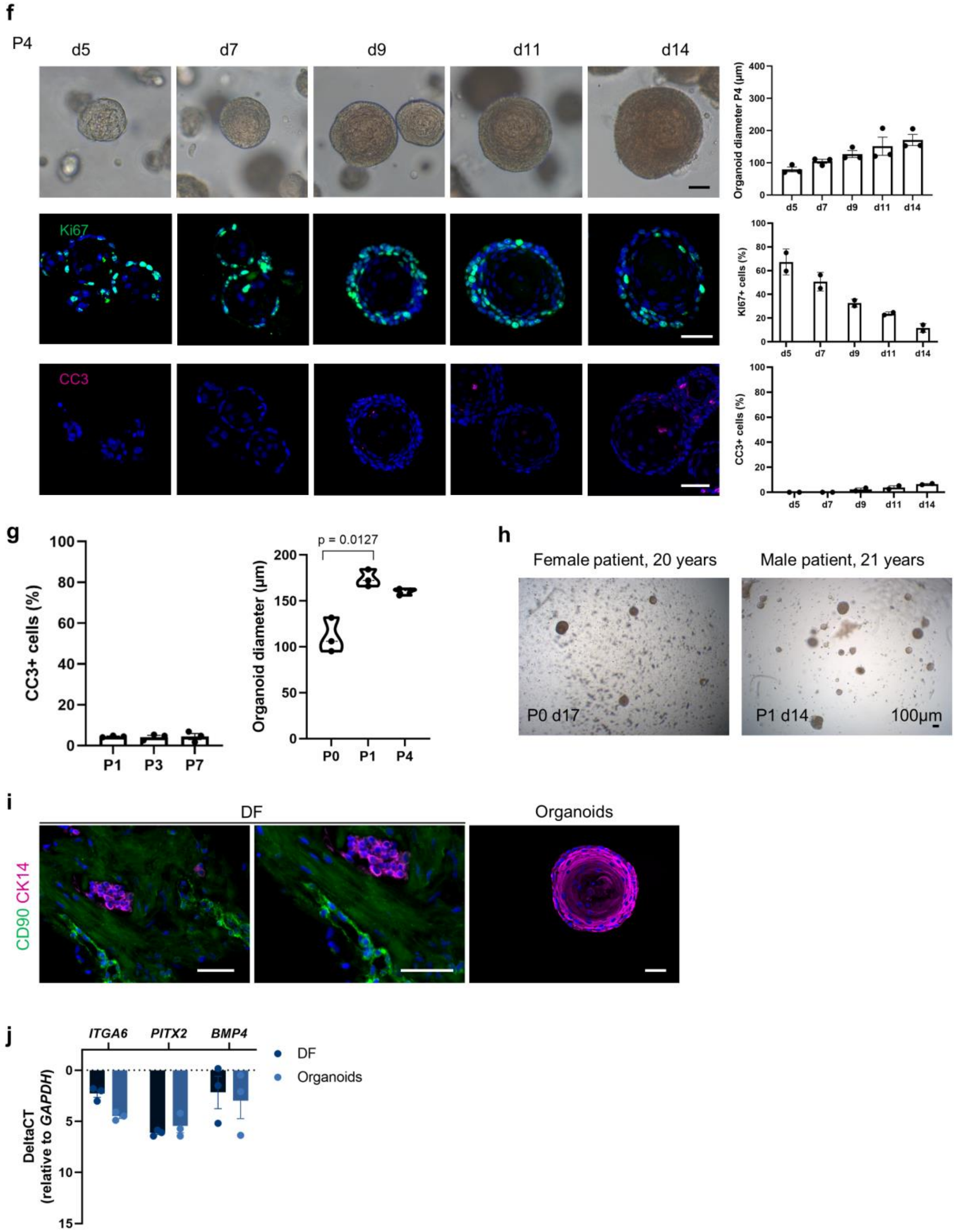

Supplementary Figure 1 Establishment of organoids from human dental follicle

a Brightfield images of the development of organoid structures (P0; day 14) after seeding dissociated dental follicle (DF) in the medium as indicated (see text). **b** Organoids growing out from DF-derived cell clusters (top) or from single cells (bottom) in tooth organoid medium (TOM; passage 0, P0; d, day). **c** Histological (H&E) analysis of DF. Boxed area is enlarged. Arrows indicate Epithelial Cell Rests of Malassez (ERM). **d** Brightfield and epifluorescence pictures of $eGFP^+$ and $eGFP^-$ cells generated from

dissociated organoids and cultured as mixture in TOM (P6; day 0 and day 14). Arrow indicates a fluorescent ($eGFP^+$) organoid and arrowheads point to non-fluorescent ($eGFP^-$) organoids. **e** Brightfield image of organoid culture after seeding of the dissociated DF tissue in TOM (P0; day 14), showing attachment of spindle-formed mesenchymal cells at the bottom of the culture plate (arrows), which are lost at passaging, being not present anymore in the first passage (P1; day 14) Consecutive magnifications are indicated by black and blue boxes, respectively. **f** Progressing organoids' development in TOM during a single-passage (P4) 14-day culture period showing brightfield pictures, organoid diameters (mean ± SEM; n=3 biological replicates), and proportions of proliferative and apoptotic events as quantified through KI67 and cleaved caspase-3 (CC3) immunostaining, respectively (dots indicate biological replicates; n=2). **g** Proportion of apoptotic ($CC3^+$) cells and diameters of day-14 organoids over different passages (mean ± SEM; n=3 biological replicates). **h** Organoids derived from DF of erupted wisdom teeth from patients as indicated. **i** Immunofluorescence staining for markers as indicated in primary DF tissue and organoids (P0, day 14). DAPI (blue) was used to label nuclei. **j** Gene expression levels (relative to *GAPDH*) of indicated markers in DF and organoids (P1) (mean ± SEM; n=3 biological replicates). Scale bars: 50µm, unless indicated otherwise.

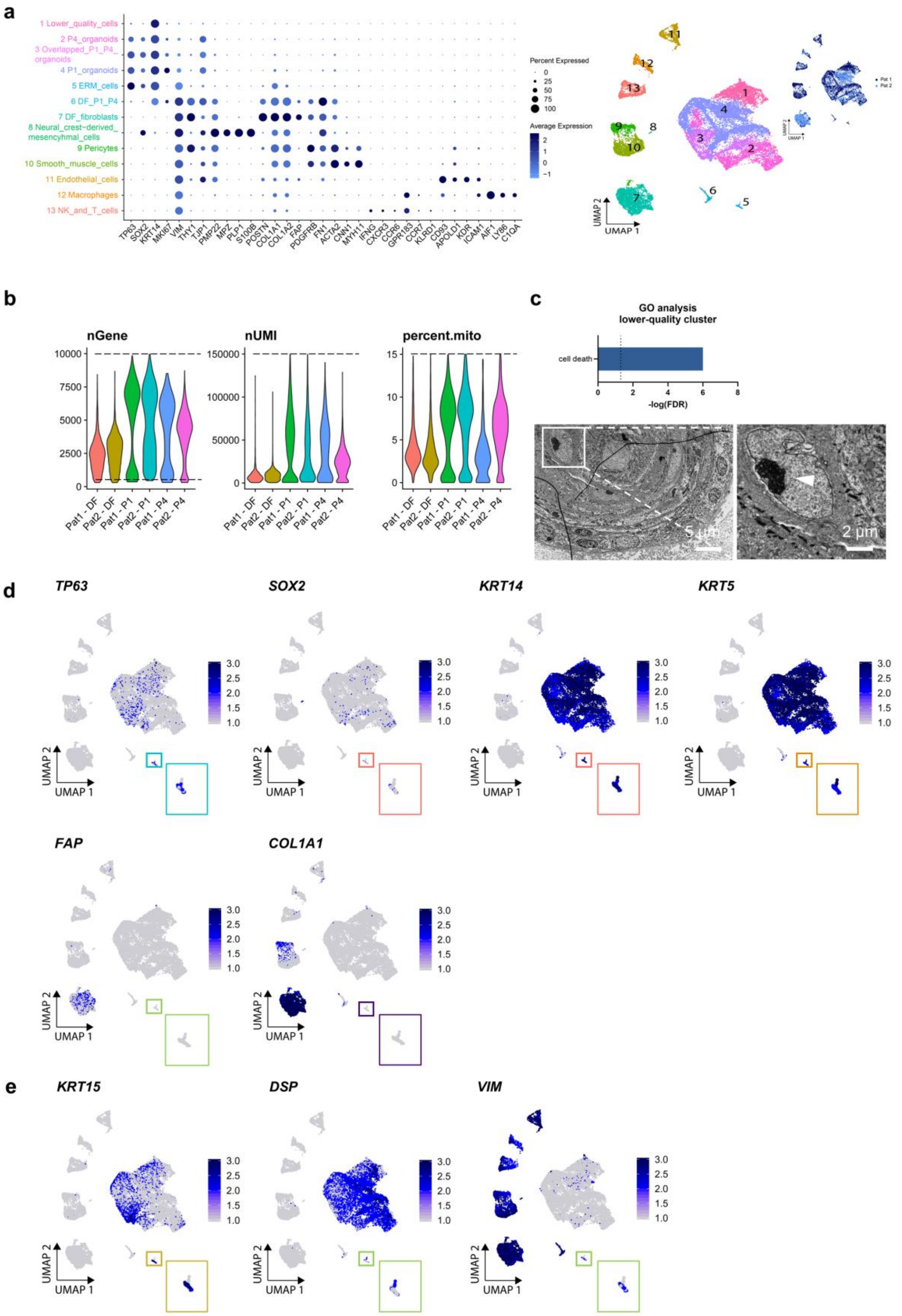
a
1 Lower_quality_cells
2 P4_organoids
3 Overlapped_P1_P4_organoids
4 P1_organoids
5 ERM_cells
6 DF_P1_P4
7 DF_fibroblasts
8 Neural_crest-derived_mesencyhmal_cells
9 Pericytes
10 Smooth_muscle_cells
11 Endothelial_cells
12 Macrophages
13 NK_and_T_cells
TP63 SOX2 KRT14 MKI67 VIM THY1 TJP1 PMP22 MPZ PLP1 S100B POSTN COL1A1 COL1A2 FAP PDGFRB FN1 ACTA2 CNN1 MYH11 IFNG CXCR3 CCR6 GPR183 CCR7 KLRD1 CD93 APOLD1 KDR ICAM1 AIF1 LY86 C1QA
Percent Expressed
0
25
50
75
100
Average Expression
2
1
0
−1
UMAP 2
UMAP 1
b
nGene
nUMI
percent.mito
Pat1 - DF
Pat2 - DF
Pat1 - P1
Pat2 - P1
Pat1 - P4
Pat2 - P4
c
GO analysis
lower-quality cluster
cell death
-log(FDR)
5 µm
2 µm
d
TP63
SOX2
KRT14
KRT5
FAP
COL1A1
e
KRT15
DSP
VIM

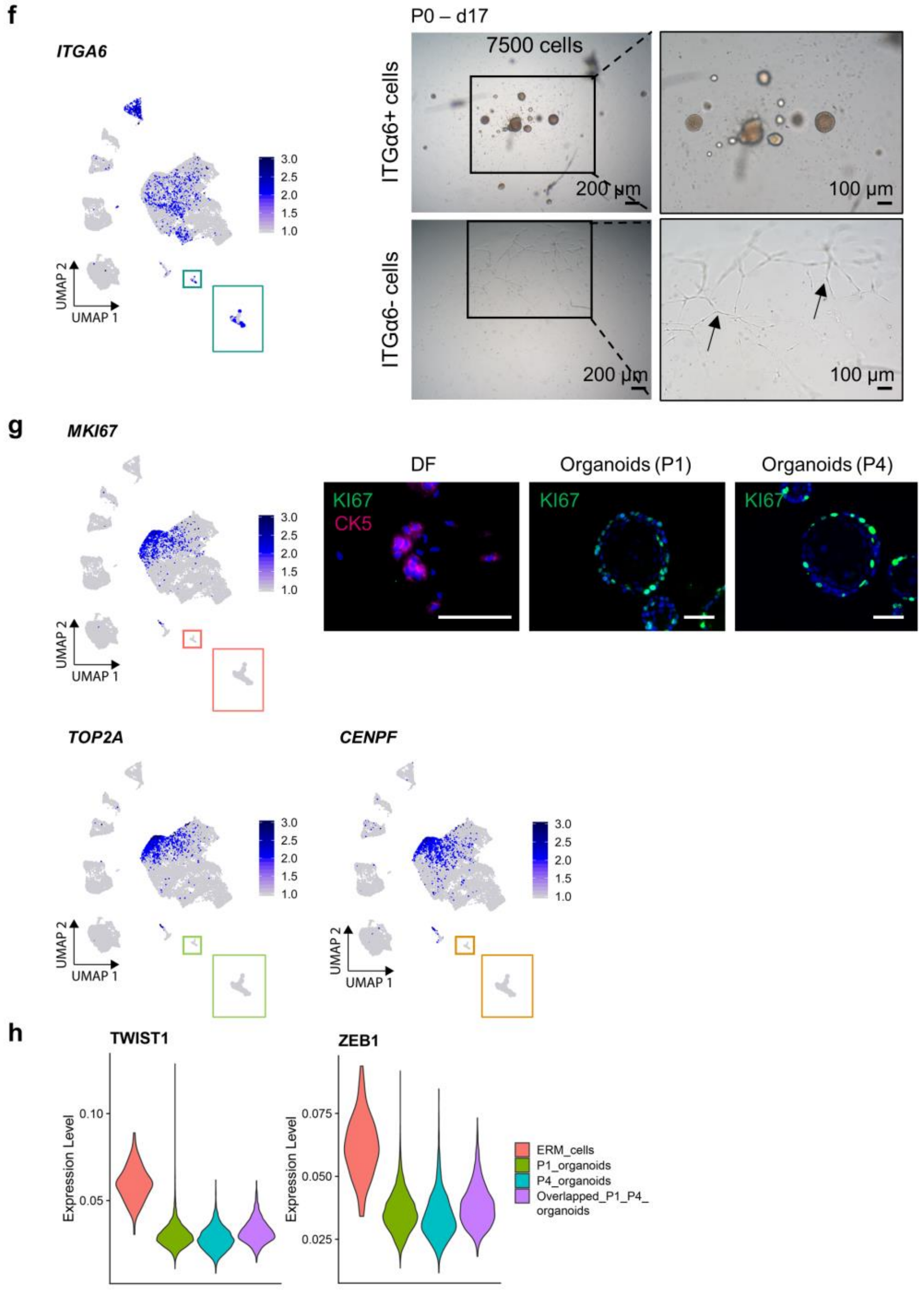

Supplementary Figure 2 Single-cell transcriptomic analysis of primary DF and corresponding organoids

a Dot plot displaying the percentage of cells (dot size) expressing indicated marker genes with average expression levels (color intensity) (see scales) of the annotated cell clusters. UMAP representation of the distinct cell clusters, and UMAP plot of the different patients (Pat). **b** Violin plots showing the distribution of the number of genes detected per cell (nGene), the total unique molecular identifier counts per cell (nUMI) and the percentage of mitochondrial content (percent.mito) per sequenced sample as indicated. Dashed lines show cut-off values (see Methods). **c** Significant (FDR ≤ 0.05) DEG-

based GO term enriched in the lower-quality cell cluster based on the top 10 DEGs. Ultrastructural (TEM) analysis of full-grown organoids (P5; day 15). Boxed area is enlarged. Arrowhead indicates an apoptotic nucleus. **d-e** Projection of indicated genes on UMAP plot. ERM cluster is enlarged at the bottom. **f** Projection of *ITGA6* expression on UMAP plot. ERM cluster is enlarged at the bottom. Brightfield pictures of organoid cultures from FACS-isolated ITGα6$^+$ or ITGα6$^-$ cells in TOM (P0; day 17). Boxed area is enlarged. Arrows indicate attached spindle-formed mesenchymal cells in the ITGα6$^-$ cell culture at the bottom of the plate. **g** Projection of indicated genes on UMAP plot. ERM cluster is enlarged at the bottom. Immunofluorescence staining of DF and (day-14) organoids for indicated markers. DAPI (blue) was used to label nuclei. **h** Violin plots displaying activity of indicated regulons in the ERM and organoid clusters. Scale bars: 50µm, unless indicated otherwise.

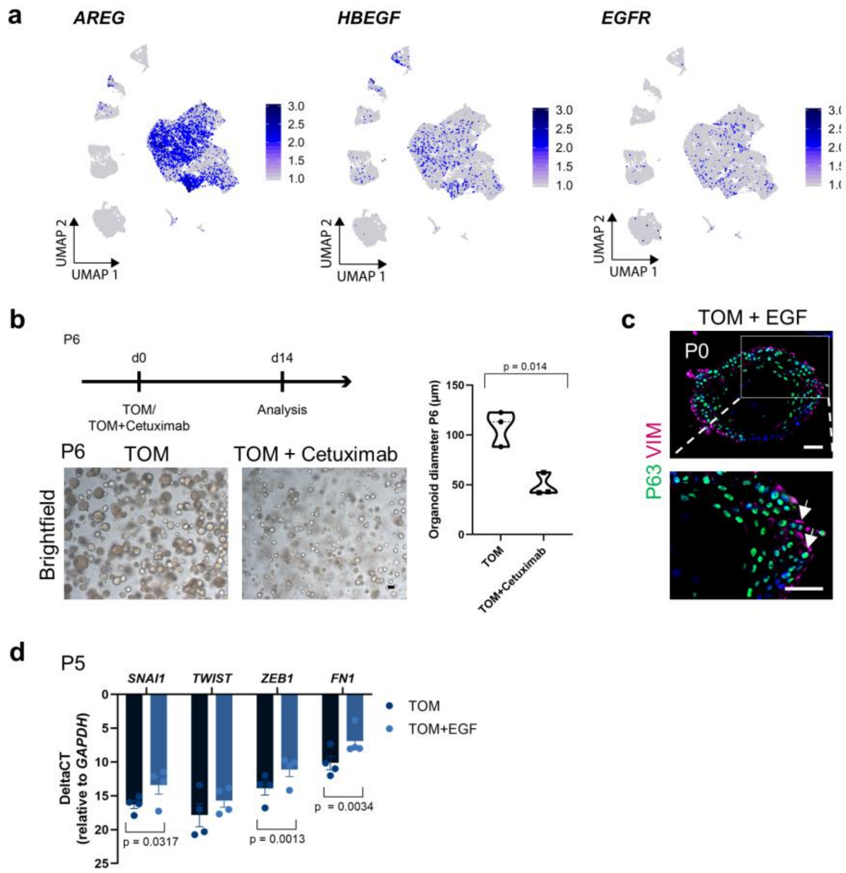

Supplementary Figure 3 Effect of EGF on tooth organoid cultures

a Projection of indicated genes on UMAP plot. **b** Timeline of experimental set-up (d, day) and brightfield pictures of organoid cultures (day 14) as indicated. Right: diameter of organoids in specified cultures (violin plot; n=3 biological replicates). **c** Immunofluorescence staining for indicated markers in organoids cultured in TOM+EGF (P0). Boxed area is enlarged. DAPI (blue) was used to label nuclei. Arrows indicate double P63$^+$VIM$^+$ cells. **d** Expression levels (relative to *GAPDH*) of indicated genes in organoids (day 14) cultured as denoted (mean ± SEM; n=4 biological replicates). Scale bars: 50µm.

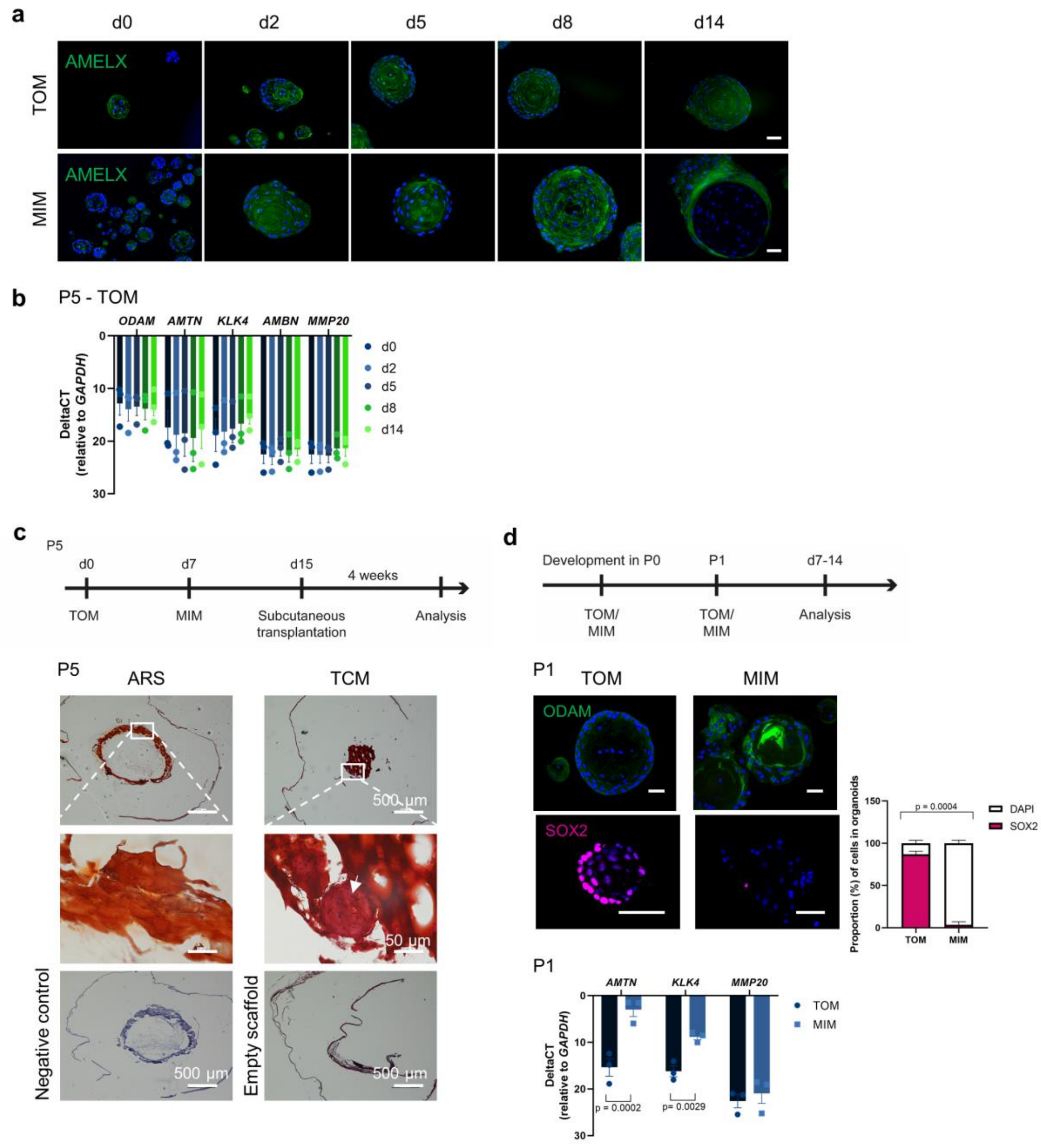

Supplementary Figure 4 Ameloblast differentiation-mimicking process in tooth organoids

a Immunofluorescence staining for AMELX in organoids cultured as denoted. DAPI (blue) was used to label nuclei. **b** Gene expression levels (relative to *GAPDH*) of indicated markers in organoids cultured in TOM at indicated time points (mean ± SEM; n=3 biological replicates). **c** Timeline of *in vivo* experimental set-up (d, day). ARS and Masson's trichrome (TCM) staining of depositions in recovered hydroxyapatite scaffolds which had been seeded with organoids before subcutaneous implantation, or only with Matrigel (empty). Boxed areas are enlarged. Negative control of ARS involves hematoxylin staining only. Arrow indicates a still discernible organoid. **d** Timeline of experimental set-up (d, day). Immunofluorescence staining for indicated markers in full-grown organoids as specified. DAPI (blue) was used to label nuclei. Quantification of $SOX2^{+}$ cells in organoids cultured as indicated (mean ± SEM;

n=3 biological replicates). Gene expression levels (relative to *GAPDH*) of indicated markers in organoids cultured as specified (mean ± SEM; n=3 biological replicates). Scale bars: 50µm, unless indicated otherwise.

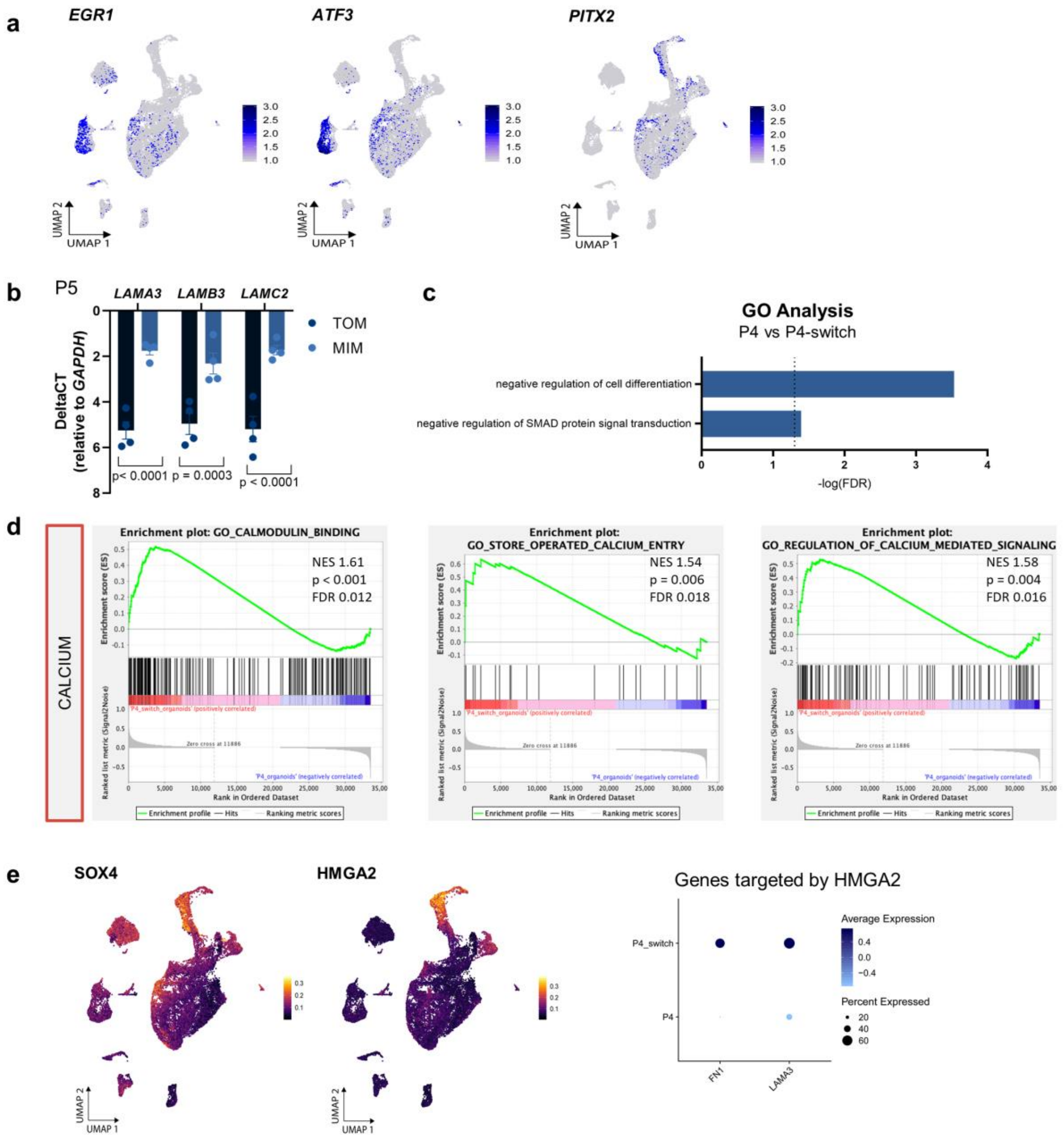
a
EGR1
ATF3
PITX2
UMAP 1
UMAP 2
b
P5
LAMA3
LAMB3
LAMC2
TOM
MIM
DeltaCT (relative to GAPDH)
p< 0.0001
p = 0.0003
p < 0.0001
c
GO Analysis
P4 vs P4-switch
negative regulation of cell differentiation
negative regulation of SMAD protein signal transduction
-log(FDR)
d
CALCIUM
Enrichment plot: GO_CALMODULIN_BINDING
NES 1.61
p < 0.001
FDR 0.012
Enrichment plot: GO_STORE_OPERATED_CALCIUM_ENTRY
NES 1.54
p = 0.006
FDR 0.018
Enrichment plot: GO_REGULATION_OF_CALCIUM_MEDIATED_SIGNALING
NES 1.58
p = 0.004
FDR 0.016
Enrichment score (ES)
Ranked list metric (Signal2Noise)
Rank in Ordered Dataset
Enrichment profile
Hits
Ranking metric scores
e
SOX4
HMGA2
Genes targeted by HMGA2
P4_switch
P4
FN1
LAMA3
Average Expression
Percent Expressed

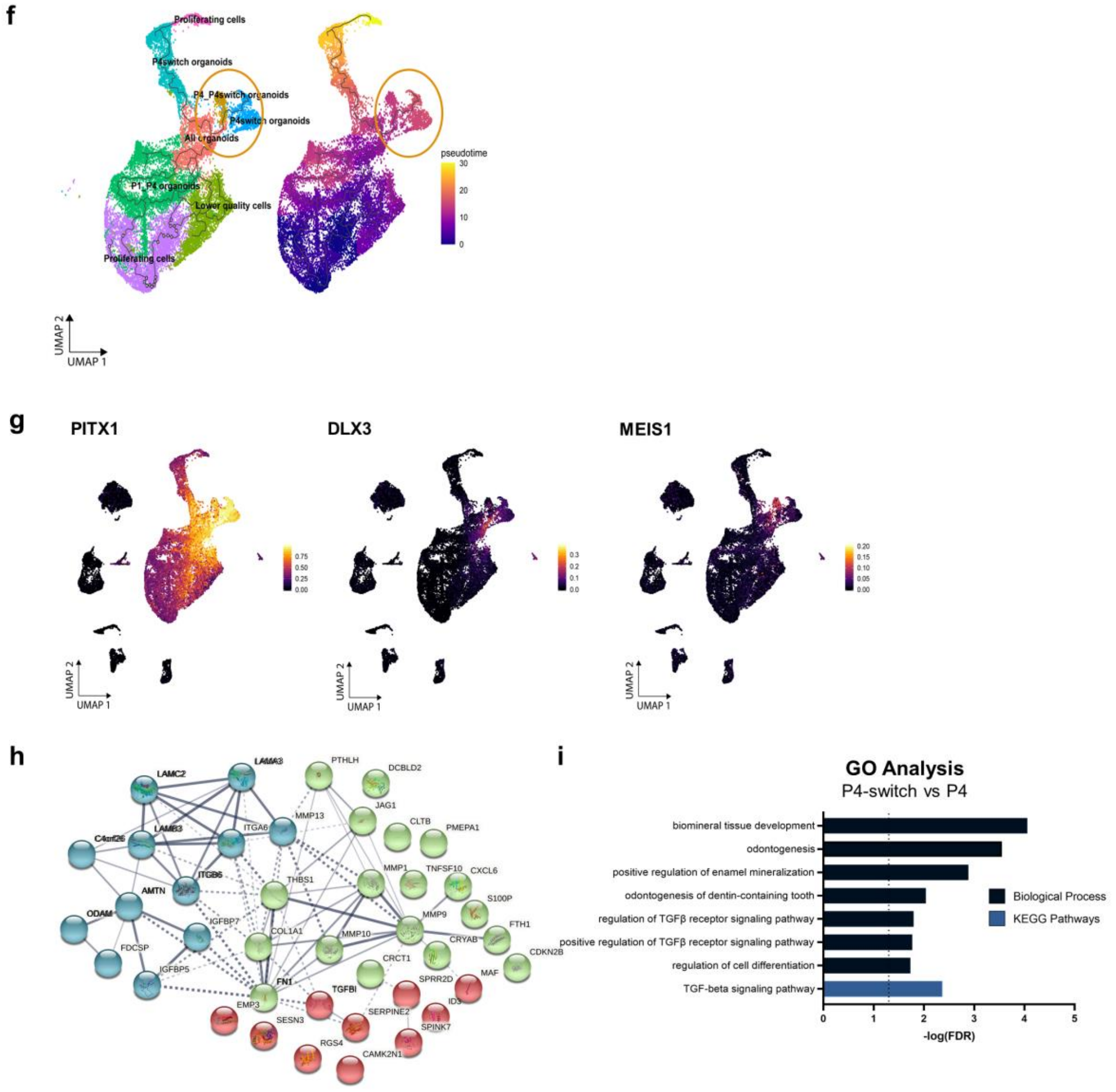

Supplementary Figure 5 Analysis of scRNA-seq data of tooth organoids driven into amelogenesis-resembling differentiation

a Projection of indicated genes on the integrated UMAP plot (see Figure 5a). **b** Expression levels (relative to *GAPDH*) of indicated genes in organoids cultured as specified (mean ± SEM; n=4 biological replicates). **c** Significant (FDR ≤ 0.05) DEG-based GO terms enriched in P4 *versus* P4-switch organoids. **d** DEG-based GSEA plots of the indicated hallmarks in P4-switch *versus* P4 organoids. Normalized enrichment score (NES-), and p- and FDR-values are listed. **e** Indicated regulons projected on the integrated UMAP plot. Dot plot of predicted HMGA2 regulon target genes in P4 and P4-switch organoids. **f** Pseudotime projected onto the integrated UMAP plot. Encircled area indicates a subcluster of potential transitional stage. **g** Indicated regulons projected on the integrated UMAP plot. **h** STRING protein-protein interaction network generated from the top 40 DEGs in P4-switch *versus* P4 organoids, predicting associations between proteins (nodes). The cluster analysis was subdivided in three colors by kmeans. Thickness of connecting line indicates confidence of interaction. Genes

specifically described in the text are highlighted in bold. **i** Significant (FDR ≤ 0.05) DEG-based GO terms enriched in top 40 DEGs of P4-switch *versus* P4 organoids by Biological Process and KEGG Pathway analysis.

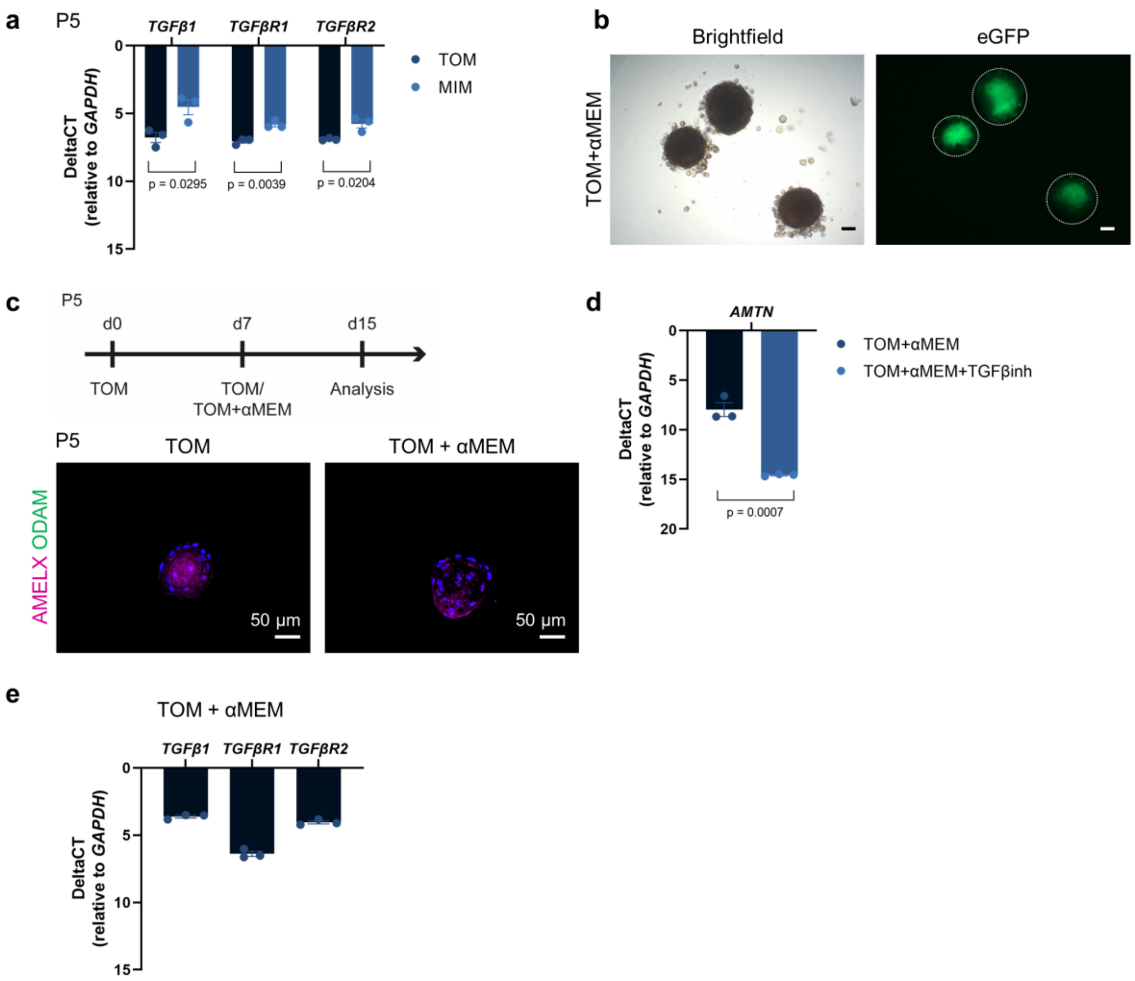

Supplementary Figure 6 Effect of TGFβ on differentiation in tooth organoids and assembloids

a Gene expression analysis of indicated TGFβ pathway components in organoids cultured as denoted. Expression is normalized to expression of *GAPDH*. Data are mean ± SEM of n=3 biological replicates. **b** Brightfield and fluorescent (eGFP) images of assembloids comprising dental epithelial (organoid-derived) cells and mesenchymal cells (DPSCs; marked by eGFP), cultured in TOM+αMEM. **c** Time-line of experimental set-up (d, day). Immunofluorescence staining for indicated markers of organoids cultured as denoted. DAPI (blue) was used to label the nuclei. Scale bar: 200µm, unless indicated otherwise. **d-e** Gene expression levels (relative to *GAPDH*) of indicated TGFβ pathway components in assembloids cultured in TOM+αMEM (mean ± SEM; n=3 biological replicates).

SUPPLEMENTARY TABLES

Supplementary Table 1 Tooth organoid medium (TOM)

Product	Concentration	Supplier	Catalogue Number
Serum-free defined medium		Thermo Fisher Scientific	For composition, see Supplementary Table 4
A83-01	0.5µM	Sigma-Aldrich	SML0788
B27 (without vitamin A)	2%	Gibco	12587-010
Cholera Toxin	100ng/ml	Sigma-Aldrich	c8052-.5mg
FGF10	100ng/ml	Peprotech	100-26
FGF2 (= basic FGF)	20ng/ml	R&D Systems	234-FSE-025
FGF8	200ng/ml	Peprotech	AF-100-25
L-glutamine	2mM	Gibco	25030024
IGF-1	100ng/ml	Peprotech	100-11
N2	1%	Gibco	17502-048
N-acetyl L-cysteine	1.25mM	Sigma-Aldrich	A7250
Nicotinamide	10mM	Sigma-Aldrich	N0636
Noggin	100ng/ml	Peprotech	120-10C
RSPO1	200ng/ml	Peprotech	120-38
SB202190	10µM	Biotechne (Tocris)	1264
SHH	100ng/ml	R&D Systems	464-SH-200
WNT3A	200ng/ml	R&D Systems	5036-WN-500

Supplementary Table 2 Overview of patient-derived organoid lines

Patient-derived organoid line	Age (year)	Sex
**Organoid_1**	18	Female
**Organoid_2**	15	Female
**Organoid_3**	16	Female
**Organoid_4**[a]	18	Male
**Organoid_5**	19	Male
**Organoid_6**	18	Female
**Organoid_7**[a]	15	Female
**Organoid_8**	16	Male
**Organoid_9**	16	Female
**Organoid_10**	17	Male
**Organoid_11**	14	Female
**Organoid_12**	15	Male
**Organoid_13**	15	Female
**Organoid_14**	17	Female
**Organoid_15**	15	Female
**Organoid_16**	17	Female
**Organoid_17**	15	Male
**Organoid_18**	15	Female
**Organoid_19**	19	Female
**Organoid_20**[b]	15	Male
**Organoid_21**	17	Male
**Organoid_22**[b]	18	Female
**Organoid_23**	15	Female
**Organoid_24**	15	Female
**Organoid_25**	19	Male
**Organoid_26**	17	Male
**Organoid_27**	17	Male
**Organoid_28**	17	Male
**Organoid_29**	17	Female
**Organoid_30**	15	Female

[a]For *in vivo* transplantation
[b]For scRNA-seq analysis

Supplementary Table 3 Mineralization-inducing medium (MIM)

Product	Concentration	Supplier	Catalogue Number
Keratinocyte serum-free medium (KBM-2)		Lonza	CC-3103
Calcium	0.09mM	Thermo Fisher Scientific	AC349610250
EGF	1ng/ml	R&D systems	236-EG-200
Bovine pituitary extract	50µg/ml	Thermo Fisher Scientific	13028014

Supplementary Table 4 Serum-free defined medium (SFDM; pH 7.3)

Name	Concentration	Supplier	Catalogue Number
Sterile H2O			
DMEM 1:1 F12 without Fe	16.8 g/L	Invitrogen	074-90715A
Transferrin	5 mg/L	Serva	36760.01
Insulin from bovine pancreas	5mg/L	Sigma-Aldrich	I6634
Penicillin	35 mg/L	Sigma-Aldrich	P3032
Streptomycin	50 mg/L	Sigma-Aldrich	S6501
Ethanol absolute, ≥99.8% (EtOH)	600µL/L	Fisher Chemical	E/0650DF/15
Catalase from bovine liver	50µL/L	Sigma-Aldrich	C100
NaHCO3	1 g/L	Merck	106329
Bovine serum albumin (BSA)	5 g/L	Serva	47330.03

Supplementary Table 5 Antibodies used for immunohistochemical/immunofluorescence staining

**Primary antibodies**				
**Antigen**	**Host**	**Supplier**	**Clone or Catalogue Number**	**Dilution**
AMELX	mouse	Santa Cruz	sc-365284	1:100
CC3	rabbit	Sigma	AB3623	1:100
CD44	mouse	Abcam	ab34485	1:200
CD90	rabbit	Abcam	ab133350	1:100
CK5	rabbit	Bioledgend	Poly19055	1:100
CK14	mouse	Thermo Fisher Scientific	LL002	1:200
CK19	mouse	Dako	M772	1:50
KI67	mouse	BD Bioscience	556003	1:100
ODAM	rabbit	Proteintech	16509-1-AP	1:200
P63	rabbit	Abcam	ab124762	1:1000
SOX2	rabbit	Abcam	ab92494	1:2000
VIM	mouse	Dako	V9	ready-to-use

**Secondary antibodies**				
**Antigen**	**Host**	**Supplier**	**Catalogue Number**	**Dilution**
mouse IgG (Alexa 488)	donkey	Thermo Fisher Scientific	A-21202	1:1000
mouse IgG (Alexa 555)	donkey	Thermo Fisher Scientific	A-31570	1:1000
rabbit IgG (Alexa 488)	donkey	Thermo Fisher Scientific	A21206	1:1000
rabbit IgG (Alexa 555)	donkey	Thermo Fisher Scientific	A-31572	1:1000

Supplementary Table 6 Primers used for qPCR

Gene	Forward primer	Reverse primer
AMBN	CCTTGCAGGAAGGAGAACTG	CTGGGAGTGATGGACCTTGT
AMTN	AGCAGGAGGAGCAGGTGTAA	CCAAATTCGAGGCAGCTTAG
BMP4	CTGGTCTTGAGTATCCTGAGCG	TCACCTCGTTCTCAGGCATGCT
CALB2	GATCCTGCCAACCGAAGAGAAC	CGATGTAGCCACTCCTGTCTGT
CASP6	AAACCAGCACGATGTGCCA	CACCTCAGTTATGTTGGTGTCC
COL3A1	TGGTCTGCAAGGAATGCCTGGA	TCTTTCCCTGGGACACCATCAG
FN1	CCATCGCAAACCGCTGCCAT	AACACTTCTCAGCTATGGGCTT
GAPDH	GGTATCGTGGAAGGACTCATGAC	ATGCCAGTGAGCTTCCCGTTCAG
ITGA6	GGCGGTGTTATGTCCTGAGTC	AATCGCCCATCACAAAAGCTC
KLK4	GGAACTCTTGCCTCGTTTCTGG	AGCGGGTCATAGAGCTTACTGC
LAMA3	TGCTCAACTACCGTTCTGCC	TCCAGTTCTTTTGCGCTTTGT
LAMB3	GCAGCCTCACAACTACTACAG	CCAGGTCTTACCGAAGTCTGA
LAMC2	TACAGAGCTGGAAGGCAGGATG	GTTCTCTTGGCTCCTCACCTTG
MDM2	GGCAGGGGAGAGTGATACAGA	GAAGCCAATTCTCACGAAGGG
MMP20	TCCATCCCTGACCTCTGTGACT	AGTGAACCTGCCGTCTCCAGAA
MSH2	AGTCAGAGCCCTTAACCTTTTTC	GAGAGGCTGCTTAATCCACTG
NCAD	CCTCCAGAGTTTACTGCCATGAC	GTAGGATCTCCGCCACTGATTC
ODAM	CAGGCCAAGTTGATCCCTTA	GAGGTTGTTCCCAGGGTAG
PITX2	CAGCGGACTCACTTTACCAG	ATTCTTGAACCAAACCCGGAC
POSTN	TGCCCAGCAGTTTTGCCCAT	CGTTGCTCTCCAAACCTCTA
SNAI1	TCGGAAGCCTAACTACAGCGA	AGATGAGCATTGGCAGCGAG
STIM1	CACTCTTTGGCACCTTCCACGT	CTGTCACCTCGCTCAGTGCTTG
TWIST1	GCCAGGTACATCGACTTCCTCT	TCCATCCTCCAGACCGAGAAGG
ZEB1	TTACACCTTTGCATACAGAACCC	TTTACGATTACACCCAGACTGC

Legends for Supplementary Datasets

Dataset 1. Cell type markers. Most discriminating genes per annotated cell type. Gene names (Gene), p values (p_val), average log fold change expression (avg_logFC), percentage of cells expressing the indicated gene in the cell type of interest (pct.1) and in all other cells together (pct.2), FDR-adjusted p value (p_val_adj) and cell type name are presented in the columns. DF, dental follicle; ERM, Epithelial Cell Rests of Malassez; NK, natural killer cells.

Dataset 2. Differentially expressed gene (DEG) analysis of ERM *versus* P1 organoids. The columns represent: gene names (Gene), p values (p_val), average log fold change expression (avg_logFC; positive values indicate higher and negative values lower expression in ERM *versus* P1 organoids), percentage of cells expressing the indicated gene in the ERM (pct.1) and in the P1 organoids (pct.2), and the FDR-adjusted p value (p_val_adj).

Dataset 3. Differentially expressed gene (DEG) analysis of ERM *versus* P4 organoids. The columns represent: gene names (Gene), p values (p_val), average log fold change expression (avg_logFC; positive values indicate higher and negative values lower expression in ERM *versus* P4 organoids), percentage of cells expressing the indicated gene in the ERM (pct.1) and in the P4 organoids (pct.2), and the FDR-adjusted p value (p_val_adj).

Dataset 4. Gene ontology (GO) analysis of DEGs between different cell type clusters. **a** GO analysis of genes upregulated in ERM *versus* P1 organoids. **b** GO analysis of genes upregulated in ERM *versus* P4 organoids. **c** GO analysis of genes upregulated in P1 organoids *versus* ERM. **d** GO analysis of genes upregulated in P4 organoids *versus* ERM. **e** GO analysis of genes upregulated in P4 organoids *versus* P4-switch organoids. **f** GO analysis of genes upregulated in P4-switch organoids *versus* P4 organoids. Columns represent: GO term (GO biological process), number of genes involved in specified GO term in the reference list (REFLIST), number of genes involved in specified GO term in uploaded DEG list (Count), number of genes expected in specified GO term based on the reference list (Expected), over- (+) representation of the GO-term (Over-representation), fold enrichment of the number of genes observed in the uploaded DEG list (Fold Enrichment), p value, FDR and -$\log_{10}$(FDR).

Dataset 5. Differentially expressed gene (DEG) analysis in P1 organoids *versus* P4 organoids. The columns represent: gene names (Gene), p values (p_val), average log fold change expression (avg_logFC; positive values indicate higher and negative values lower expression in P1 organoids *versus* P4 organoids), percentage of cells expressing the indicated gene in the P1 organoids (pct.1) and in the P4 organoids (pct.2), and the FDR-adjusted p value (p_val_adj).

Dataset 6. Differentially expressed gene (DEG) analysis in P4 organoids *versus* P4-switch organoids. The columns represent: gene names (Gene), p values (p_val), average log fold change expression (avg_logFC; positive values indicate higher and negative values lower expression in P4 organoids *versus* P4-switch organoids), percentage of cells expressing the indicated gene in the P4 organoids (pct.1) and in the P4-switch organoids (pct.2), and the FDR-adjusted p value (p_val_adj).

Dataset 7. Protein-protein interactions of top 40 DEGs of P4-switch organoids *versus* P4 organoids analyzed by STRING. **a** Protein 1 (node1) interacts with protein 2 (node2), id of protein 1 (node1_string_id), id of protein 2 (node2_string_id), experimentally determined protein interaction (experimentally_determined_interaction), protein annotations (database_annotated), textmining evidence extracted from abstract of scientific literature (automated_textmining), combined protein-protein interaction score (combiner_score). **b-c** GO analysis of Biological Processes and KEGG pathways of top 40 DEGs of P4-switch organoids *versus* P4 organoids. Columns represent: GO term ID, term description, observed gene count, background gene count, strength, FDR, matching proteins in the network (IDs and labels).
